# Streambed Microbial Activity and Its Spatial Distribution in Two Intermittent Stream Networks

**DOI:** 10.3390/microorganisms14010071

**Published:** 2025-12-29

**Authors:** Andrielle L. Kemajou Tchamba, Charles T. Bond, Brett A. Nave, Claire Utzman, Jerald Ibal, Delaney M. Peterson, C. Nathan Jones, Carla L. Atkinson, Erin C. Seybold, Robert J. Ramos, Amy J. Burgin, Lydia H. Zeglin, Yaqi You, Ken Aho, Kevin A. Kuehn, Colin R. Jackson

**Affiliations:** 1 Department of Biology, University of Mississippi, University, MS 38677, USA; akemajou@go.olemiss.edu; 2 School of Biological, Environmental and Earth Sciences, University of Southern Mississippi, Hattiesburg, MS 39406, USA; 3 Division of Biology, Kansas State University, Manhattan, KS 66506, USA; 4 Department of Biological Sciences, Idaho State University, Pocatello, ID 83209, USA; 5 Department of Biology, University of Alabama, Tuscaloosa, AL 35487, USA; 6 Department of Biological and Ecological Engineering, Oregon State University, Corvallis, OR 97331, USA; 7 Kansas Geological Survey, Department of Geology, University of Kansas, Lawrence, KS 66045, USA; 8 The Environmental Data Science Innovation & Inclusion Lab, University of Colorado, Boulder, CO 80309, USA; 9 Department of Ecology, Evolution and Organismal Biology, Iowa State University, Ames, IA 50011, USA; 10 Department of Environmental Resources Engineering, College of Environmental Science and Forestry, The State University of New York, Syracuse, NY 13210, USA

**Keywords:** microbial enzyme activity, spatial variation, streambed habitat, intermittent stream, enzyme stoichiometry

## Abstract

Headwater streams comprise almost 90% of global river networks, and their microorganisms play critical roles in organic matter decomposition and nutrient cycling. These functions, however, are affected by recurrent drying and rewetting. This study examined spatial variation in microbial enzyme activity tied to organic carbon degradation (β-glucosidase, phenol oxidase, and peroxidase) and nitrogen (N-acetylglucosaminidase) and phosphorus (phosphatase) mineralization in water, epilithic biofilm, leaf litter, and sediment in two intermittent streams: Gibson Jack Creek (Idaho, USA) and Pendergrass Creek (Alabama, USA), representing different climactic and physiographic settings. Microbial activity was greater in Gibson Jack Creek, where the activity of leaf litter enzymes varied along the stream network, and there were strong correlations in microbial activity between different stream habitats. Microbial activity in Pendergrass Creek showed primarily within-habitat associations. Activity in water, sediment, and biofilm showed broader spatial heterogeneity in both stream networks. Ratios of microbial activity (enzyme stoichiometry) suggested that microbial communities in both systems were primarily limited by carbon and phosphorus, although there was more spatial variation in nitrogen limitation, particularly in water and sediment at Pendergrass Creek and in biofilm at Gibson Jack Creek. These findings underscore the spatial heterogeneity and environmental sensitivity of microbial processes in intermittent streams.

## 1. Introduction

Organic material in headwater streams is decomposed and mineralized by heterotrophic microorganisms, which drive nutrient cycling [1]. Microbial communities in these streams degrade organic matter by releasing extracellular enzymes, with microbial enzymatic activity being influenced by factors such as temperature, pH, and oxygen and organic matter availability [2,3]. Broader-scale factors, such as land cover, anthropogenic impacts, and climate change, can further shape stream microbial activity, affecting nutrient cycling and water quality [4,5]. Headwater streams account for almost 90% of the global river network, and microbial processes in these systems drive downstream water quality and ecosystem services [6,7]. However, about 60% of headwater streams experience intermittent flow, a condition expected to become more prevalent with increasing water abstraction and climate change [8]. Increased flow intermittency will further disrupt the transport and transformation of water, energy, dissolved and suspended materials, and organisms within stream networks [9,10].

Intermittent streams are increasingly recognized for their ecological importance, particularly as the number and extent of intermittent streams expand under climate change and water resource development [11,12]. These streams are highly complex systems, with multiple factors controlling the spatiotemporal distribution of flow [8]. Intermittent streams, like perennial streams, can be highly variable in drainage area, hydrology, geomorphology, and substrate characteristics both within networks and between systems or watershed [13,14]. However, the repeated cycles of drying and rewetting set them apart from perennial systems. As a result, these periodic transitions between aquatic and terrestrial phases, with streambeds of intermittent streams acting as hotspots for microbial activity and biogeochemical processes [15], significantly influence carbon and nutrient cycling [11,16]. Variations in water availability, along with shifts in redox state, temperature, and water retention capacity of the different streambed habitats, impact the composition and function of stream microbial communities, influencing stream ecosystem processes at multiple spatial scales [17,18].

Microorganisms play vital roles in stream ecosystems, and their activity is shaped by the quantity and quality of organic matter as well as prevailing environmental conditions [3,19]. These factors are influenced by the timing, quantity, and characteristics of allochthonous inputs of organic matter into streams, which are, in turn, governed by watershed features such as vegetation, climate, geomorphology, and hydrology [20,21]. Drying can drastically alter the ecological, chemical, and physical conditions within streams [22], fragmenting the stream network and generating spatial heterogeneity through disconnected flow paths and diverse microhabitats. While watershed-level features are relatively consistent across time outside of rare, large-scale meteorological events, drying means that stream mesohabitats and microhabitats are variable at fine spatial and temporal scales, requiring their microbial communities to respond to changing conditions [8]. Microorganisms can respond swiftly at the physiological level, but are also influenced by broader-scale drivers such as organic matter availability and quality [23,24], and the disconnected nature of intermittent stream systems means that variability in microbial activity can have long-lasting ecological consequences [9].

Patterns in microbial extracellular enzyme activity serve as fingerprints of rates of organic matter degradation and nutrient mineralization [24]. Ratios in the activity of different enzymes (enzyme stoichiometry) can indicate the limitation of the microbial community by particular elements [25,26]. Although microbial processes have been studied in a variety of streams [26,27,28,29], spatial patterns in this activity—particularly across multiple habitats and streams—remain poorly understood. Streams are heterogeneous, with diverse microbial habitats that can disproportionately influence the flow of energy and nutrients. Such dynamics may be especially important in intermittent streams, which form a mosaic of wet and dry habitats along the network continuum. Despite this, there is limited knowledge of how stream intermittency relates to microbial activity across watersheds with different climates, physiography, and land use [22]. Cross-system studies are, therefore, critical for identifying how and why microbial processes vary regionally [20]. Existing findings range from minimal variation in microbial activity between different stream habitats to pronounced heterogeneity in activity across stream networks [30,31,32,33], highlighting the roles of small-scale variation and the watershed-level influences of climate and geology [24,34].

In this study, we compared microbial enzyme activity in four different streambed habitats within two intermittent streams from distinct ecogeographical regions of the United States. Our aim was to evaluate how microbial processes are distributed spatially within streams (across habitats and along the flow path) and between stream networks that differ in climate, hydrology, and watershed characteristics. Because these processes are coupled to spatial heterogeneity in organic matter availability, hydrological connectivity, and physicochemical conditions at local (habitat) and broader (watershed) scales, we hypothesized that microbial enzyme activity would show spatial variation both within each stream and between streams. Such differences in microbial activity over different scales should be expected, but quantifying them is critical for identifying patterns or constraints on microbial function across intermittent stream networks. We used enzyme stoichiometry to assess abiotic constraints on microbial communities in both systems, allowing us to evaluate whether common or stream-specific factors regulate microbial processes in intermittent streams. While studies of single systems are important, cross-scale comparisons of microbial activity in intermittent streams in different climatic and hydrological settings provide a better framework for distinguishing common drivers of microbial processes from system-specific controls.

## 2. Materials and Methods

### 2.1. Study Sites

**Figure 1:**
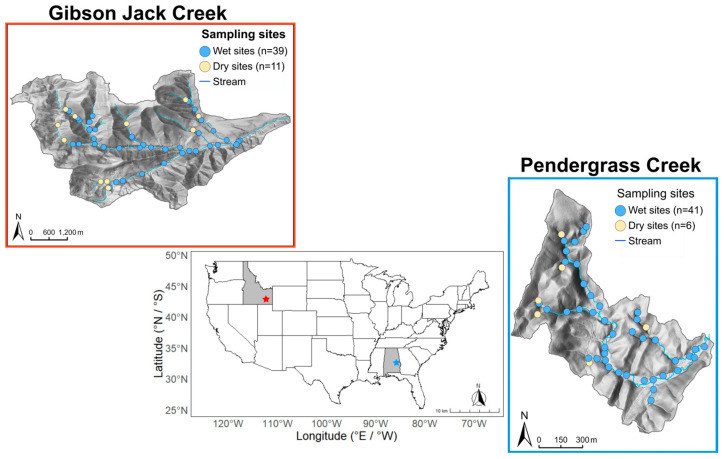
Sites (circles) in Gibson Jack Creek (red star), Idaho (blue star), and Pendergrass Creek, Alabama, USA, that were assayed for microbial extracellular enzyme activity. Light blue circles indicate wet sites at the time of sampling, while light yellow circles represent dry sites. Enzyme activity was measured at each site in water (if present), epilithic biofilm, leaf litter, and sediment.

This study was conducted in two intermittent streams: Gibson Jack Creek (Idaho, USA) and Pendergrass Creek (Alabama, USA) (Figure 1). Gibson Jack Creek is a headwater stream in the Caribou-Targhee National Forest (near Pocatello, Idaho), draining 25.5 km^2^ of steep, high-relief watershed, with elevations ranging from 1555 to 2130 m. It is a tributary of the Portneuf River, which flows into the Snake River and ultimately, into the Columbia River [35]. The region has a semi-arid steppe climate with a mean annual temperature ranging from below 0 to 30 °C and a mean annual precipitation of 614.5 mm. The south-facing slope of the drainage area is dominated by sagebrush, juniper, and grasses, while the north-facing slope mostly supports coniferous trees. The watershed consists of quartzite and shale bedrock in the north and limestone in the south, with stream substrates primarily composed of silt loam, sandy clay loam, and gravel [36].

Pendergrass Creek is located in the Talladega National Forest (Cleburne County, Alabama) [37] and drains a 0.92 km^2^ of mixed coniferous and deciduous forest over an elevation range of 345–456 m. It contributes to Choccolocco Creek, a tributary of the Coosa River. The region has a humid subtropical climate with mean annual temperatures ranging from 5 to 25.3 °C, and mean annual precipitation of 1400 mm. The watershed is a mixed coniferous–deciduous forest, dominated by pines (loblolly, longleaf) and oaks (mixed red and white species), and the bedrock is metamorphic, characterized by fractured quartzite, metasiltstone, and phyllite with primarily silt loam soil texture [38]. The stream bedrock consists of saprolite cobbles, gravel, and sand substrates [39].

### 2.2. Sample Collection and Environmental Conditions at Study Sites

Sample collection and processing have been described in detail in Plont et al. [38]. Samples of four different microbial habitats were collected from 47 sites in the Pendergrass Creek network from 9 to 10 June 2022, and from 50 sites in the Gibson Jack Creek network a year later from 27 to 30 June 2023 (Table 1, Figure 1). While sampling each system at the same time would have been ideal for stream–stream comparisons, the logistics of sampling and site accessibility meant that we were required to sample in different years to sample each stream at the same time of year. The four habitats were surface water (where present) and three types of streambed habitat (leaf litter, epilithic biofilm, and sediment). At each site, a transect spanning the full wetted channel width (0.27–3.8 m at Gibson Jack Creek; 0.4–2.5 m at Pendergrass Creek) was established, and three evenly spaced locations along each transect were sampled. Samples from the three locations on the transect were pooled to form a composite sample for each habitat type at each site. Surface water samples (120 mL) were collected using a sterile syringe at each of the three locations along the transect and combined in a sterile Nalgene bottle. The composite water sample was mixed, and 40 mL was transferred to a sterile 50 mL centrifuge tube for later assays. For leaf litter, a submerged decomposing leaf (typically *Acer glabrum* or *Betula nigra* at Gibson Jack Creek, and *Liriodendron tulipifera*, or *Quercus* sp., at Pendergrass Creek, depending on the prevailing vegetation) was collected from each location on the transect, cut using sterile scissors, and half of each leaf was placed into a sterile 15 mL centrifuge tube. For sediment, the top 2 cm of sediment from each of the three locations along the transect was collected using a sterile 50 mL centrifuge tube as a coring device. Sediment samples were pooled in the 50 mL centrifuge tube, and the composite sample was mixed using a sterile Scoopula. For biofilm, a small (fist-sized or less) rock was collected from each transect location, and approximately 25 cm^2^ of its upper surface was scraped using a sterile wire brush and rinsed with sterile deionized water to create a biofilm slurry. Slurries from the three locations on each transect were combined and mixed into a sterile 50 mL tube, and 10 mL of the resulting mixture was transferred to a sterile 15 mL centrifuge tube. The total scraped rock area at each site was calculated by summing the upper surface area of all the rocks sampled. All samples were kept on ice for transport to the laboratory and then stored at −20 °C until analysis.

Environmental and physicochemical variables were measured at each site, using approaches described in previous studies [38,40,41]. Site characteristics of slope, elevation, topographic wetness index (TWI), drainage area, and distance from the stream outlet were derived from a 2 m resolution digital elevation model (DEM) using the Whitebox package version 2.4.0 [42]. Canopy cover was estimated in the field using a densiometer. Water presence or absence was determined using a Stream Temperature Intermittency Conductivity (STIC) sensor deployed to each site approximately three weeks prior to sampling [43]. At sites with surface water, composite water samples were homogenized, filtered (pre-ashed 0.7-μm glass fiber, Whatman GF/F), and analyzed for concentrations of non-purgeable organic carbon (NPOC) using a TOC-V Carbon analyzer (Shimadzu, Columbia, MD, USA). Major anion and cation concentrations were measured on acidified, filtered (0.45-μm PVDF filter, VWR) samples using ion chromatography (Dionex Integrion HPIC, Agilent Technologies, Santa Clara, CA, USA) or induced coupled plasma-atomic emission spectrometry (Horiba Ultima 2, Horiba Instruments, Houston, TX, USA).

Concentrations of ammonium, orthophosphate, and nitrate were determined for stream sediments by shaking 10 g of sediment with 10 mL degassed deionized water for 1 h at 1.33× *g*, followed by centrifugation at 3220× *g* for 45 min and filtration through a 0.42 μm Whatman GF/F filter. Extracts were analyzed using colorimetric microplate assays: the indophenol blue method for ammonium (630 nm), the molybdenum blue method for orthophosphate (880 nm), and the vanadium chloride/Griess method for nitrate (540 nm) [44,45]. Nutrient concentrations were determined from standard curves and expressed as μg g^−1^ sediment dry weight. The pH of sediment extracts was measured using a SevenCompact pH meter (Mettler Toledo, Columbus, MD, USA). Watershed characteristics and physicochemical parameters relevant to this study are available in the HydroShare repository (links provided in the Data Availability Statement Section), and a summary of measured variables is shown in Table 2.

### 2.3. Assays of Microbial Extracellular Enzyme Activity

The activities of five microbial extracellular enzymes associated with organic matter decomposition (β-glucosidase (BG), phenol oxidase (POX), peroxidase (PER)), and organic phosphorus (phosphatase (P)) and nitrogen (N-acetylglucosaminidase (NAG)) mineralization were determined as described in Kemajou Tchamba et al. [40] and following the protocols of Jackson et al. [46]. Activities of β-glucosidase, phosphatase, and N-acetylglucosaminidase in water and biofilms were assayed using fluorescent 4-methylumbelliferone (MUB)-linked substrates, while activities in leaf litter and sediment were determined with colorimetric 4-nitrophenyl (pNP)-linked substrates. Activities of phenol oxidase and peroxidase were assayed colorimetrically only for leaf litter and sediment using L-3,4-dihydroxyphenylalanine (DOPA). Water and biofilm samples were thawed, vortexed, and distributed into microplate wells with appropriate MUB standards or substrates. Fluorescence readings were recorded every 5 min for 30 min, and enzyme activity was calculated as substrate consumption rates in μmoles h^−1^mL^−1^ for water and μmoles h^−1^cm^−2^ for biofilm. For leaf litter and sediment, slurries were prepared by homogenizing the sample in sterile water, and subsamples of each slurry were incubated with the appropriate substrate for 1–3 h. Enzyme activities were calculated as substrate consumption rates (μmoles h^−1^g^−1^ dry weight). Moisture content and dry mass of leaf litter and sediment samples were determined by mass loss of subsamples dried at 70 °C for 48 h. Ash-free dry mass was then determined by combusting the dried subsamples at 500 °C for 2 h.

### 2.4. Microbial Nutrient Limitation Based on Enzyme Stoichiometry

Potential nutrient limitation was determined from stoichiometric analysis of enzyme activity, which infers microbial resource limitation from hydrolase and oxidase activities involved in carbon and nutrient acquisition [26,47]. The fraction of N-acetylglucosaminidase activity in the sum of N-acetylglucosaminidase and phosphatase activity (i.e., NAG/[NAG + P]) was plotted on the x-axis, while the fraction of β-glucosidase activity in the sum of β-glucosidase and N-acetylglucosaminidase activity (i.e., BG/[BG + NAG]) was plotted on the y-axis, with 0.5 as a threshold for identifying C, N, or P limitation. To refine the assessment of C limitation in leaf litter and sediment, the y-axis was modified to represent overall carbon-degrading enzyme activity, including the activity of enzymes degrading both labile (BG) and recalcitrant (phenol oxidase, POX, and peroxidase, PER) carbon sources, relative to NAG (i.e., mean BG and POX and PER/[NAG + mean BG and POX and PER]). This framework classified potential microbial resource limitation into four categories: C and N co-limited, C and P co-limited, P-limited, or N-limited.

### 2.5. Statistical Analysis

Statistical analyses were conducted in R version 4.4.2 [48]. For each analysis, the effective sample sizes (*n*) were determined separately for each stream and habitat based on the number of observations with complete data (Table 1). Dry sites were excluded from any water enzyme and water chemistry analyses but retained for the three other streambed habitats, where samples were collected regardless of surface water presence. Differences in microbial enzyme activities between streams were tested using Wilcoxon rank-sum tests with false discovery rate (FDR) correction. Cross-habitat and functional relationships among enzymes were evaluated using Kendall’s Tau correlations (corrplot v0.95 [49]) and visualized as heatmaps. Spatial variation in activity within habitats was assessed using generalized additive models (GAMs; mgcv v1.9-3) with a Tweedie distribution, including habitat as a fixed effect and in-stream distance and spatial coordinates as smooth terms [50]. Partial redundancy analysis (RDA; vegan v2.6-10) was used to relate microbial enzyme activities to water chemistry and environmental variables. Only significant and non-collinear predictors were retained through correlation and variance inflation factor threshold (VIF < 0.8) analyses [51]. Geographically weighted regression (GWR; GWmodel v2.4-1) was used to quantify spatially varying relationships between enzyme activity and environmental drivers, using in-stream distance networks to account for hydrologic connectivity [52,53]. Site-level coefficients were pooled to obtain stream-specific effect sizes, and meta-analytic contrasts compared predictor influences between streams. Additional packages included sf v1.0-19 and ggplot2 v3.5.1. Statistical significance was set at *p* < 0.05.

## 3. Results

### 3.1. Spatial Distribution of Extracellular Enzyme Activity

**Figure 2:**
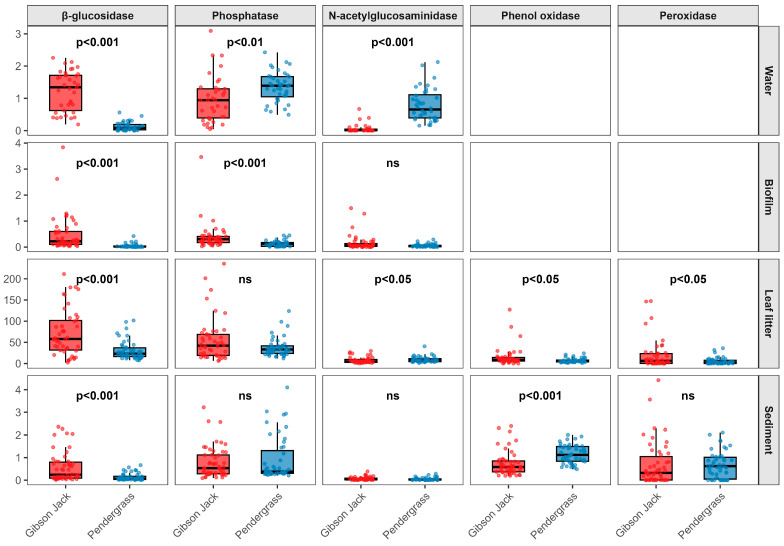
Microbial extracellular enzyme activity (points) in stream water (μmol h^−1^ mL^−1^), biofilm (μmol h^−1^ cm^−2^), leaf litter (μmol h^−1^ g^−1^), and sediment (μmol h^−1^ g^−1^) across 50 sites in Gibson Jack Creek (red) and 47 sites in Pendergrass Creek (blue). Boxes show the interquartile range (IQR), with the median indicated by the horizontal line and whiskers extending to 1.5 × IQR. Differences in enzyme activity were assessed using the Wilcoxon rank-sum test with false discovery rate (FDR) correction. Effective sample sizes (*n*) varied by habitat and enzyme based on the presence of each habitat at each sample site. In water, *n* = 35–37 at Gibson Jack Creek and *n* = 40 at Pendergrass Creek; in biofilm, *n* = 48 and *n* = 42, respectively; and in leaf litter and sediment, *n* = 50 at Gibson Jack Creek and *n* = 47 at Pendergrass Creek.

**Figure 3:**
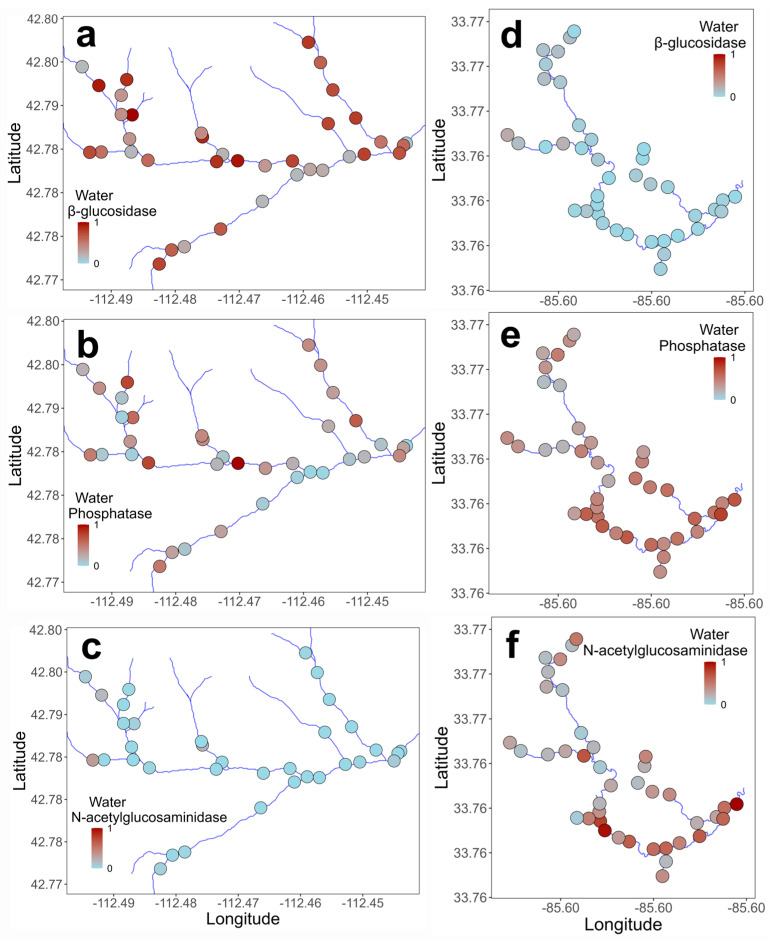
Spatial distribution of microbial extracellular enzyme activity in water across 37 and 40 sites in Gibson Jack Creek, Idaho, and Pendergrass Creek, Alabama, USA. Enzyme activity has been z-score-transformed, with red indicating the highest activity and blue indicating the lowest activity for that enzyme. Activity is shown for Gibson Jack Creek (**a**–**c**) and Pendergrass Creek (**d**–**f**). Actual β-glucosidase activity (μmol h^−1^ mL^−1^) ranged from 0.2 to 2.3 (Gibson Jack Creek) and 0–0.6 (Pendergrass Creek). Phosphatase activity ranged from 0.05 to 3.1 (Gibson Jack Creek) and 0.5–2.4 (Pendergrass Creek), and N-acetylglucosaminidase activity ranged from 0 to 0.7 (Gibson Jack Creek) and 0.2–2.1 (Pendergrass Creek).

**Figure 4:**
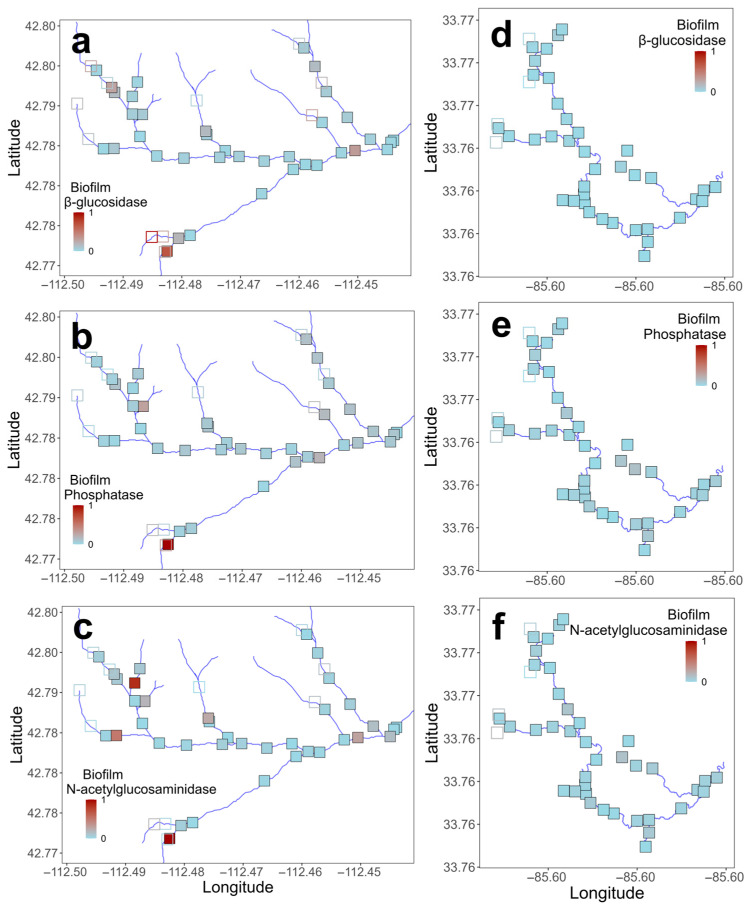
Spatial distribution of microbial extracellular enzyme activity in biofilm across 48 and 42 sites in Gibson Jack Creek, Idaho, and Pendergrass Creek, Alabama, USA. Enzyme activity has been z-score-transformed, with red indicating the highest activity and blue indicating the lowest activity for that enzyme. Activity is shown for Gibson Jack Creek (**a**–**c**) and Pendergrass Creek (**d**–**f**), with filled symbols indicating wet sites and open symbols indicating dry sites. Actual β-glucosidase activity (μmol h^−1^ cm^−2^) ranged from 0.03 to 3.8 (Gibson Jack Creek) and 0–0.4 (Pendergrass Creek). Phosphatase activity ranged from 0.03 to 3.5 (Gibson Jack Creek) and 0.01–0.4 (Pendergrass Creek), and N-acetylglucosaminidase activity ranged from 0 to 1.5 (Gibson Jack Creek) and 0.003–0.3 (Pendergrass Creek).

Microbial enzyme activity varied both within and between the two stream networks, with differences in activity by stream, enzyme, and microbial habitat (Table 3; Figure 2, Figure 3 and Figure 4; Supplementary Information (SI): Table S1). Enzyme activities in water were generally higher in Gibson Jack Creek than in Pendergrass Creek, except for β-glucosidase, which showed no difference in activity between the two streams (Table 3, Figure 2). There was no significant spatial variability in microbial activity in water based on in-stream distance, although the activities of phosphatase and N-acetylglucosaminidase varied with geographic location in Pendergrass Creek, with activity increasing downstream (*p* < 0.05; Figure 3e,f; Table S1). Enzyme activities in biofilm were also higher in Gibson Jack Creek than in Pendergrass Creek, other than for N-acetylglucosaminidase (Table 3, Figure 2). Spatial variation in biofilm microbial activity was minimal, although biofilm β-glucosidase activity showed significant geographic variation in both watersheds, with higher activity in stream tributaries than the main channel (Figure 4a,d; *p* < 0.05; Table S1).

**Figure 5:**
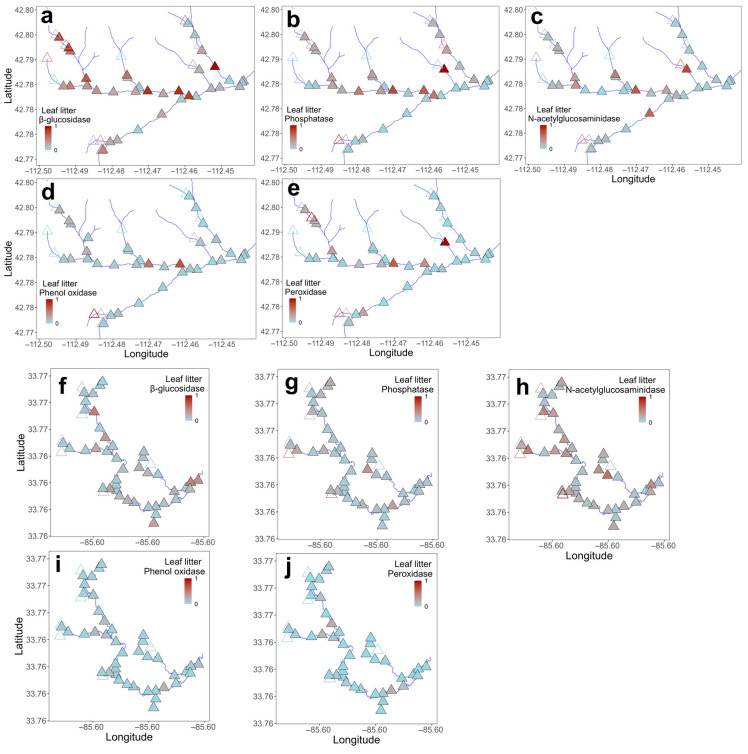
Spatial distribution of microbial extracellular enzyme activity in leaf litter across 47 and 47 sites in Gibson Jack Creek, Idaho, and Pendergrass Creek, Alabama, USA. Enzyme activity has been z-score-transformed, with red indicating the highest activity and blue indicating the lowest activity for that enzyme. Activity is shown for Gibson Jack Creek (**a**–**e**) and Pendergrass Creek (**f**–**j**), with filled symbols indicating wet sites and open symbols indicating dry sites. At Gibson Jack Creek, actual enzyme activities (μmol h^−1^ g^−1^) ranged from 2.3 to 211.3 for β-glucosidase, 6.2–235.6 for phosphatase, 0.28–29.69 for N-acetylglucosaminidase, 0–127.3 for phenol oxidase, and 0–147.3 for peroxidase. Corresponding ranges for Pendergrass Creek were 7.6–101.8, 11.9–123.9, 1.3–40.6, 0.9–24, and 0–36.04.

**Figure 6:**
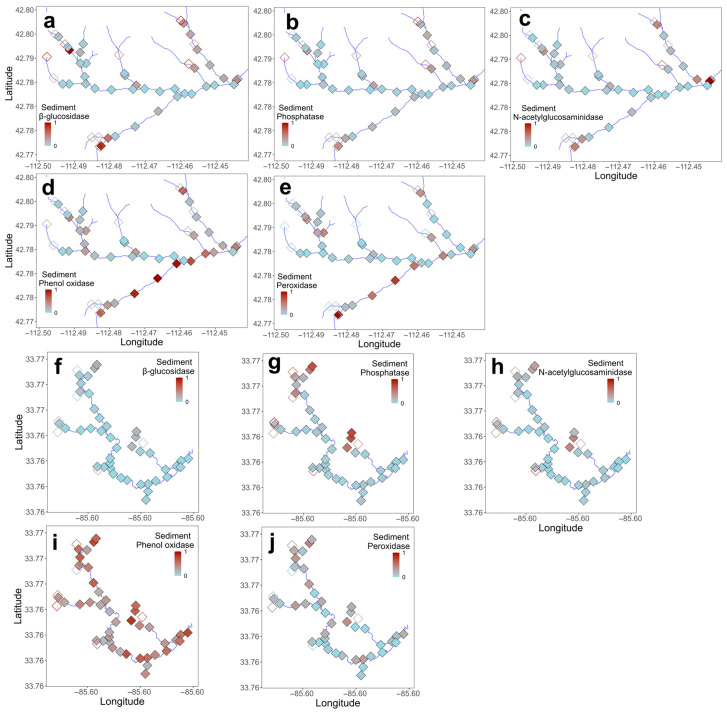
Spatial distribution of microbial extracellular enzyme activity in sediment across 50 and 47 sites in Gibson Jack Creek, Idaho, and Pendergrass Creek, Alabama, USA. Enzyme activity has been z-score-transformed, with red indicating the highest activity and blue indicating the lowest activity for that enzyme. Activity is shown for Gibson Jack Creek (**a**–**e**) and Pendergrass Creek (**f**–**j**), with filled symbols indicating wet sites and open symbols indicating dry sites. At Gibson Jack Creek, actual activities (μmol h^−1^ g^−1^) ranged from 0.01 to 2.4 for β-glucosidase, 0.1–3.2 for phosphatase, 0.004–0.4 for N-acetylglucosaminidase, 0.2–2.4 for phenol oxidase, and 0–4.4 for peroxidase. Corresponding ranges for Pendergrass Creek were 0.005–0.7, 0.2–4.1, 0–0.3, 0.5–2.01, and 0–2.1.

Activities of β-glucosidase, phenol oxidase, and peroxidase in leaf litter were greater in Gibson Jack Creek than in Pendergrass Creek, while leaf litter N-acetylglucosaminidase activity was higher in Pendergrass Creek, and phosphatase activity in leaf litter did not differ between the two streams (Table 3, Figure 2). Spatial variation in leaf litter microbial activity was significant in Gibson Jack Creek, where the activities of phosphatase and phenol oxidase often differed between adjacent sample sites, while the activities of both oxidases differed across the stream network (Figure 5a,b,g,h; *p* < 0.05; Table S1). There was no significant spatial variation in leaf litter enzyme activity in Pendergrass Creek (Figure 5d–f,i,j; *p* > 0.05; Table S1). Sediment enzyme activities were broadly similar across the two streams, although β-glucosidase activity was higher in Gibson Jack Creek and phenol oxidase activity was higher in Pendergrass Creek (Table 3, Figure 2). There was significant spatial variation in the sediment activity of the three hydrolases in Gibson Jack Creek, but not in the activity of the oxidative enzymes (Figure 6a–c; *p* < 0.05; Table S1). No spatial variation was detected in sediment microbial enzyme activity in Pendergrass Creek (Figure 6d–f,i,j; *p* > 0.05; Table S1).

### 3.2. Relationships Between Environmental Variables and Extracellular Enzyme Activity

The activities of different microbial enzymes were correlated within and across habitats in both streams, with Gibson Jack Creek showing broader cross-habitat associations and Pendergrass Creek showing stronger within-habitat correlations (Figure S1). In Gibson Jack Creek, the activity of hydrolases in all habitats was positively correlated with each other and with the activity of the oxidative enzymes in leaf litter and sediment (Figure 3a; T = 0.27–0.7; *p* < 0.05). Activities of the two oxidative enzymes in leaf litter and sediment were also correlated with each other (T = 0.43–0.46; *p* < 0.01). There were negative correlations between biofilm phosphatase activity and leaf litter β-glucosidase activity, and between activity of the leaf litter hydrolases and sediment phenol oxidase (T = −0.35–(−0.43); *p* < 0.01). In Pendergrass Creek, correlations in enzyme activity were strongest within habitats, with sediment enzymes showing the strongest correlations with each other (Figure 3b; T = 0.22–0.72; *p* < 0.05). The activity of enzymes in leaf litter also showed within- and between-enzyme class correlations (T = 0.24–0.72; *p* < 0.05), though cross-habitat associations were weak and limited (T = −0.24–(−0.26); *p* < 0.05). N-acetylglucosaminidase activity in water was positively correlated with phosphatase activity but negatively correlated with β-glucosidase activity (T = −0.3–0.47; *p* < 0.01), while enzyme activity in biofilms only showed positive correlations between the different enzymes (T = 0.25–0.66; *p* < 0.05).

**Figure 7:**
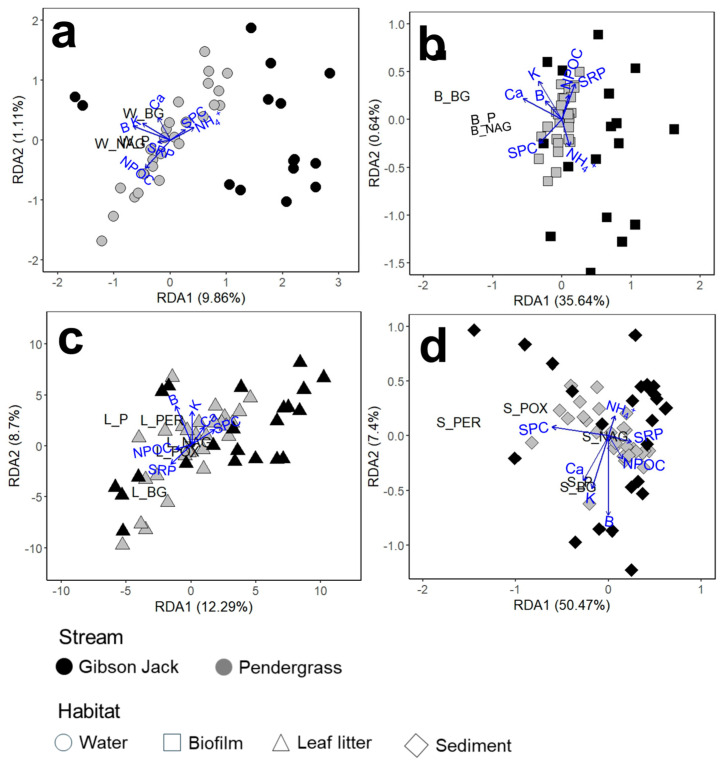
Partial redundancy analysis (pRDA) ordination showing relationships between extracellular enzyme activities and water chemistry variables in water ((**a**): circles), biofilm ((**b**): squares), leaf litter ((**c**): triangles), and sediment ((**d**): diamonds) across Gibson Jack Creek (black) and Pendergrass Creek (gray). The overall sample size for the analysis was *n* = 53, consisting of 26 sites in Gibson Jack Creek and 27 sites in Pendergrass Creek. Filled symbols indicate wet sites at the time of sampling, while unfilled symbols represent dry sites. Enzymes were β-glucosidase (BG), phosphatase (P), N-acetylglucosaminidase (NAG), phenol oxidase (POX), and peroxidase (PER). Arrows represent water chemistry variables, with direction and length indicating the strength and orientation of their correlations with enzyme activity. Axes correspond to canonical components derived from the pRDA constrained by environmental predictors, with percentage values showing the variance explained by each axis. SRP indicates soluble reactive phosphorus, SPC is specific conductivity, and POC is particulate organic carbon.

Relationships between microbial enzyme activity and water chemistry were consistently stronger at Gibson Jack Creek, where >50% of the variation in sediment enzyme activity could be explained by the measured chemical variables (Figure 7). The leading contributors to this variability were boron and organic carbon in water, calcium and potassium in biofilm, boron and soluble reactive phosphorus in leaf litter, and boron and specific conductivity in sediment. Hydrolase activities in different microbial habitats in Gibson Jack Creek showed spatial variation, which was mostly positively correlated to calcium concentration but negatively correlated to potassium and magnesium concentrations (Figure S2). Oxidase activity in leaf litter and sediment also displayed spatial variation, which was positively linked to potassium and calcium concentrations for leaf litter (Figure S2i,j), while patterns in sediment phenol oxidase activity were correlated positively with strontium and conductivity but negatively with calcium, and peroxidase activity was positively related to calcium and yet negatively related to potassium (Figure S2d,e). In Pendergrass Creek, water chemistry had weaker and less consistent relationships to spatial variation in microbial enzyme activity. Leaf litter N-acetylglucosaminidase and peroxide activities showed spatial heterogeneity, which was positively influenced by potassium concentration and conductivity (Figure S2h–j), while oxidase activity in sediment and leaf litter showed spatial variation, which was negatively influenced by the concentrations of magnesium and sodium in the water column (Figure S2d,e,i,j).

**Figure 8:**
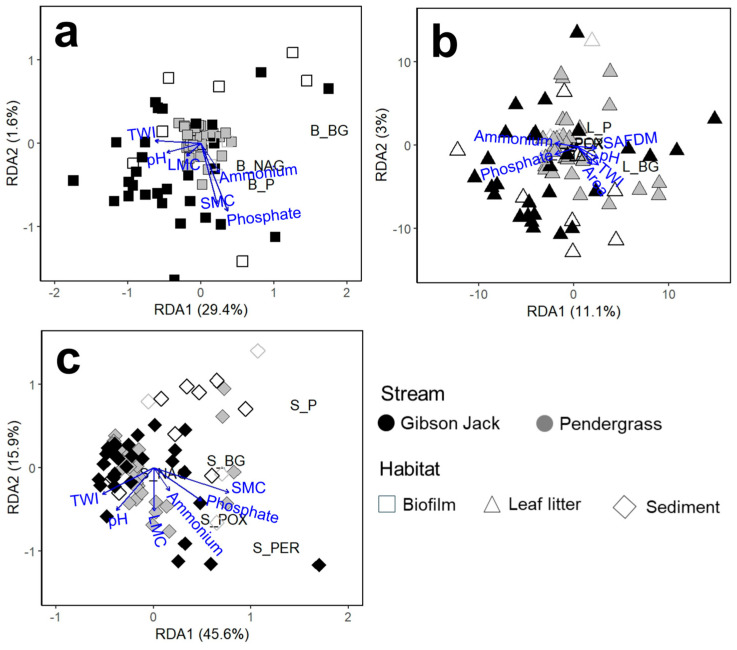
Partial redundancy analysis (pRDA) ordination showing relationships between extracellular enzyme activities and habitat and watershed features in biofilm ((**a**): squares), leaf litter ((**b**): triangles), and sediment ((**c**): diamonds) across Gibson Jack Creek (black) and Pendergrass Creek (gray). Filled symbols indicate wet sites at the time of sampling, while unfilled symbols represent dry sites. Effective sample size was *n* = 41 for Gibson Jack Creek (wet sites *n* = 32, dry sites *n* = 9) and *n* = 35 for Pendergrass Creek (wet sites *n* = 31, dry sites *n* = 4). Enzymes include β-glucosidase (BG), phosphatase (P), N-acetylglucosaminidase (NAG), phenol oxidase (POX), and peroxidase (PER). Arrows represent environmental variables, with direction and length indicating the strength and orientation of their correlations with enzyme activity. Axes correspond to canonical components derived from the pRDA constrained by environmental predictors, with percentage values showing the variance explained by each axis. TWI indicates topographic wetness index, S_AFDM and L_AFDM are sediment and leaf litter ash-free dry mass, and S_moisture and L_moisture are sediment and leaf litter moisture, respectively.

Watershed and habitat characteristics explained 61.5% of the variation in sediment microbial enzyme activity. Across habitats, the strongest predictors were sediment moisture, phosphate concentration, and topographic wetness index, with sediment phosphate concentration emerging as a consistent driver (Figure 8). In Gibson Jack Creek, sediment and biofilm hydrolase activities showed spatial variations that were positively related to elevation and drainage area, but negatively related to distance from the outlet for sediment and topographic wetness index (TWI) for biofilm (Figure S3a–c,k–m). Leaf litter hydrolase activity showed spatial patterns that were positively linked to TWI, litter organic matter content, and sediment nutrient concentrations, except for sediment phosphate concentration, which showed a negative relationship to leaf litter hydrolase activity, as well as in drainage area (Figure S3f,g). In Pendergrass Creek, hydrolase activities showed spatial variation with minimal correlations to watershed or habitat characteristics, except in leaf litter. Leaf litter β-glucosidase and phosphatase activities showed spatial variation, which was positively related to TWI, leaf litter organic matter content, and sediment ammonium and nitrate concentrations but negatively related to sediment phosphate concentration, drainage area, and elevation (Figure S3f,g). Sediment and leaf litter oxidative enzyme activities also showed spatial variations in both streams, influenced by watershed variables. Sediment oxidase activity displayed spatial patterns that were positively related to drainage area in both streams; however, other variables showed contrasting relationships. For instance, sediment oxidative enzyme activity showed spatial patterns that were driven by sediment moisture content in Gibson Jack Creek but were negatively related in Pendergrass Creek. In contrast, those spatial patterns were negatively correlated with TWI in Gibson Jack Creek but positively correlated in Pendergrass Creek (Figure S3d,e). Leaf litter oxidative enzyme activity also showed spatial variation that was positively correlated with distance from the outlet and with sediment ammonium and nitrate concentrations in Gibson Jack Creek, but showed negative correlations in Pendergrass Creek. Conversely, these spatial patterns were positively related to elevation and sediment phosphate concentration in Pendergrass Creek but negatively in Gibson Jack Creek (Figure S3i,j).

### 3.3. Enzyme Stoichiometry

**Figure 9:**
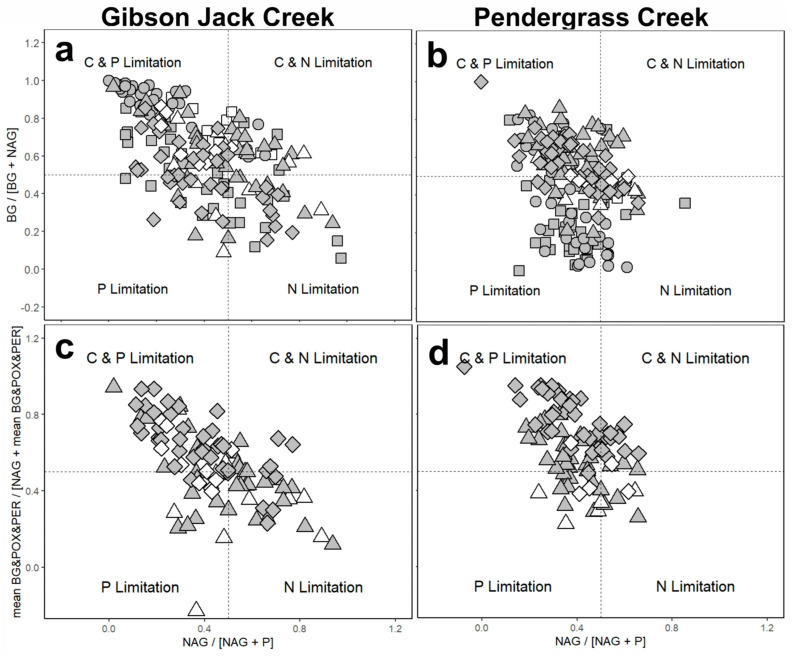
Stoichiometric analysis of microbial enzyme activity in Gibson Jack Creek, Idaho, USA (**a**,**c**) and Pendergrass Creek, Alabama, USA (**b**,**d**). Hydrolase stoichiometry is shown for Gibson Jack Creek (**a**) and Pendergrass Creek (**b**) in water (circles), biofilm (squares), leaf litter (triangles), and sediment (diamonds), and combined hydrolase–oxidase stoichiometry is shown for Gibson Jack Creek (**c**) and Pendergrass Creek (**d**) in leaf litter (triangles) and sediment (diamonds). Filled symbols indicate wet sites at the time of sampling, while unfilled symbols represent dry sites. At Gibson Jack Creek, the sample size of wet sites was *n* = 37 for water and biofilm, *n* = 35 for leaf litter, and *n* = 39 for sediment, while for dry sites it was *n* = 11 for biofilm, leaf litter, and sediment. At Pendergrass Creek, the sample size of wet sites was *n* = 40 for water, *n* = 38 for biofilm, and *n* = 41 for leaf litter and sediment, while for dry sites it was *n* = 4 for biofilm and *n* = 6 for leaf litter and sediment. Microbial nutrient limitation was categorized into four groups: C and N co-limited (top right of each panel), C and P co-limited (top left), P-limited (bottom left), and N-limited (bottom right). Dashed lines indicate the predicted 1:1:1 relationship between C-, N-, and P-acquiring enzymes. Filled symbols represent wet sites, while open symbols represent dry sites at the time of sampling. Hydrolases measured were β-glucosidase (BG), N-acetylglucosaminidase (NAG), and phosphatase (P), while the oxidases were phenol oxidase (POX) and peroxidase (PER).

Stoichiometric enzyme analysis suggested that microbial communities in water in Gibson Jack Creek were mostly co-limited by C and P, whereas those in Pendergrass Creek were generally limited by P or co-limited by C and P, with more frequent N limitation than seen in water microbial communities in Gibson Jack Creek (Figure 9). Biofilm microbial communities in Gibson Jack Creek were also generally co-limited by C and P, compared to being mostly P-limited in Pendergrass Creek, where biofilms were rarely limited by N. Microbial communities in leaf litter in Gibson Jack Creek were equally co-limited by C and P and C and N, whereas those in leaf litter in Pendergrass Creek showed similar enzyme stoichiometry to sediment in both streams, with co-limitation by C and P being the most common. N limitation was more prevalent in microbial communities in leaf litter than in sediment at Gibson Jack Creek, whereas it was more prevalent in sediment than in leaf litter at Pendergrass Creek (Figure 9).

**Figure 10:**
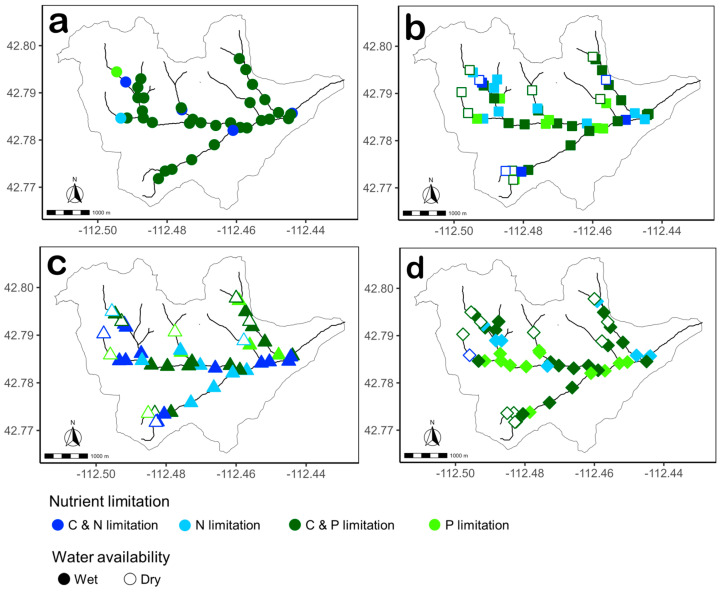
Spatial distribution of microbial nutrient limitation based on hydrolase stoichiometry in water ((**a**) circles, *n* = 37), biofilm ((**b**) squares, *n* = 48), leaf litter ((**c**) triangles, *n* = 46), and sediment ((**d**) diamonds, *n* = 50) in Gibson Jack Creek, Idaho, USA. Nutrient limitation was categorized into four groups: N-limited (light blue), P-limited (light green), C and N co-limited (dark blue), and C and P co-limited (dark green). Filled symbols represent wet sites (*n* = 37 for water and biofilm, *n* = 35 for leaf litter, and *n* = 39 for sediment) and open symbols represent dry sites (*n* = 11 for biofilm, leaf litter, and sediment) at the time of sampling.

**Figure 11:**
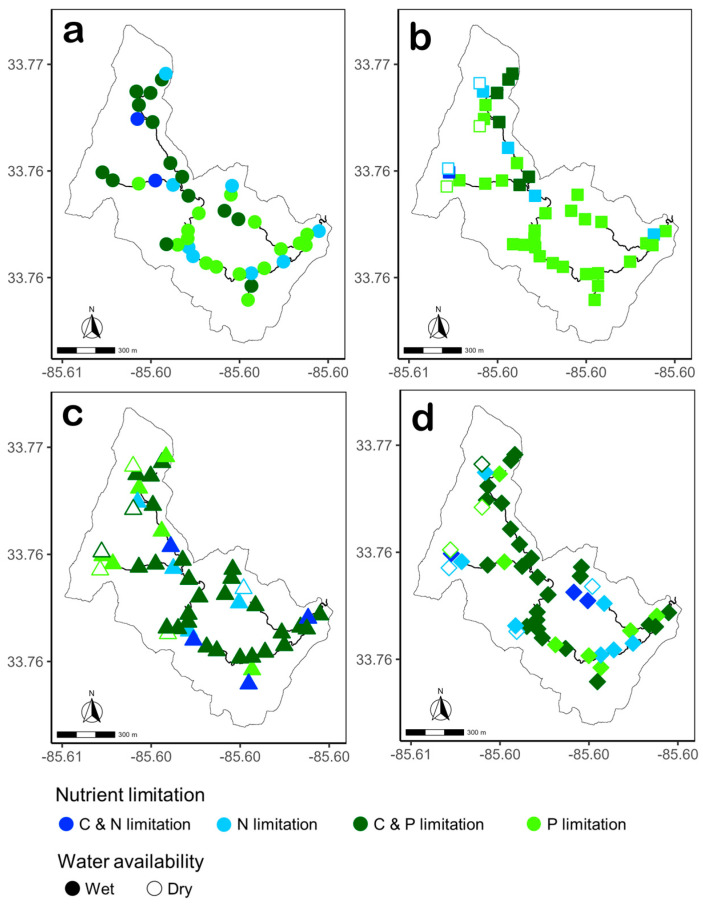
Spatial distribution of microbial nutrient limitation based on hydrolase stoichiometry in water ((**a**) circles, *n* = 40), biofilm ((**b**) squares, *n* = 42), leaf litter ((**c**) triangles, *n* = 47), and sediment ((**d**) diamonds, *n* = 47) in Pendergrass Creek, Alabama, USA. Nutrient limitation was categorized into four groups: N-limited (light blue), P-limited (light green), C and N co-limited (dark blue), and C and P co-limited (dark green). Filled symbols represent wet sites (*n* = 40 for water, *n* = 38 for biofilm, and *n* = 41 for leaf litter and sediment) and open symbols represent dry sites (*n* = 4 for biofilm and *n* = 6 for leaf litter and sediment) at the time of sampling.

The spatial distribution of nutrient limitation in each habitat differed between the two streams (Figure 10 and Figure 11). In Gibson Jack Creek, spatial patterns were minimal with no clear structure, whereas spatial patterns in nutrient limitation in Pendergrass Creek were more pronounced. In water, C and P co-limitation was widespread throughout Gibson Jack Creek, whereas C and P co-limitation in water microbial communities of Pendergrass Creek was localized upstream, with P limitation more common at downstream sites (Figure 10a and Figure 11a). Spatial distribution of nutrient limitation in biofilms in Gibson Jack Creek showed clusters of N-limited sites at confluences with tributaries, while N limitation in Pendergrass Creek biofilm communities was rare (Figure 10b and Figure 11b). Spatial patterns in the nutrient limitation of leaf litter and sediment microbial communities were limited, as these communities were mostly C and P co-limited in both streams, although there was some N limitation in some tributaries, a pattern that was more localized in Gibson Jack Creek leaf litter and Pendergrass Creek sediments (Figure 10c,d and Figure 11c,d).

## 4. Discussion

Intermittent headwater streams exhibit variation in hydrology, organic matter availability, and nutrient cycling, with the ecological responses to these changes differing between watersheds with contrasting climate and physiography [22]. Although microbial processes in these systems have received growing attention [21,54,55,56], the distribution of microbial activity between microhabitats and over entire stream networks is still poorly understood. In this study, we compared the spatial distribution of microbial extracellular enzyme activity in four streambed habitats (water, epilithic biofilm, leaf litter, and sediment) in Gibson Jack Creek (Idaho) and Pendergrass Creek (Alabama), USA. There were marked differences in the magnitude and spatial variability in microbial activity between the two streams, highlighting the importance of environmental conditions in each system as well as the heterogeneity of streambed habitat. Stoichiometry analysis of enzyme activities revealed differences in the nutrient limitation of microbial communities in different habitats and between streams.

The range of enzyme activities observed in this study was generally comparable to or higher than values previously reported for comparable habitats in other lotic systems [33,57,58]. Enzyme activity was generally higher in Gibson Jack Creek than in Pendergrass Creek, although sediment microbial enzyme activity was similar between the two systems, and sediment phenol oxidase activity was higher in Pendergrass Creek. This contrast may reflect hydrological conditions at the time of sampling: Pendergrass Creek received substantial rainfall within the 24 h prior to sampling, likely homogenizing nutrient distribution and reducing variability in microbial activity. In contrast, the absence of recent precipitation at Gibson Jack Creek may have promoted stronger nutrient localization, microbial acclimation, and enzymatic stability, resulting in higher overall enzyme activities. Studies comparing microbial enzyme activity between streams in different regions are limited, with some reporting differences in activity for all enzymes in water and sediment [33,56], and others reporting differences only for specific enzymes [57]. While differences in enzyme activity between streams in this study were quite pronounced, within each system, there was less variation in activity between sites, except for leaf litter in Gibson Jack Creek, where activities differed with in-stream distance between sites. Microbial enzyme activity in leaf litter can vary within a single-stream network, potentially accounting for variation in organic matter decomposition rates, which typically decrease from headwaters to downstream reaches [58,59,60,61]. The spatial distribution of microbial activity in other habitats varied: biofilm β-glucosidase activity showed spatial variation in both streams, phosphatase and N-acetylglucosaminidase activity in water only differed spatially in Pendergrass Creek, and the sediment hydrolases only showed spatial patterns in activity at Gibson Jack Creek. The levels of spatial heterogeneity in enzyme activity reported in other studies range from limited spatial variability in water and epilithic biofilms [29,33] to significant spatial variation in epilithic biofilms [62,63] and sediment [32]. Collectively, these results highlight the stream-to-stream specificity of enzyme activities and the strong influence of local habitat heterogeneity on patterns in microbial function [57,64,65].

In both streams, the activities of the three hydrolases (β-glucosidase, N-acetylglucosaminidase, and phosphatase) were positively correlated with each other for all habitats, while activities of the two oxidative enzymes (phenol oxidase, peroxidase) correlated with each other in leaf litter and sediment. Activities of these enzymes in different habitats were also correlated, particularly for leaf litter and sediment. A similar pattern was observed in an intermittent stream in the American Great Plains [40], suggesting a possible relationship of activity in both habitats to a common factor, such as microbial biomass, a more stable microbial community, or shared organic matter input, leading to parallel increases in enzyme activity in sediment and leaf litter [66,67]. These between-habitat correlations were more common in Gibson Jack Creek than in Pendergrass Creek, where correlations between the activity of different enzymes were common within a habitat. These patterns suggest that habitat compartments may be more strongly differentiated at Pendergrass Creek than at Gibson Jack Creek.

Water chemistry and stream organic matter quality can be influenced by the hydrological and bedrock geological processes of a watershed, such as groundwater contributions, mineral weathering, or subsurface flow paths [68,69], with streams draining metamorphic tending to have lower pH and cation concentrations than those draining limestone [70,71]. The two streams in this study followed that pattern, with water in Gibson Jack Creek (limestone) having higher pH and cation concentrations than water in Pendergrass Creek (metamorphic). Cations strongly influenced spatial patterns in enzyme activity in Gibson Jack Creek, but only minimally in Pendergrass Creek. There was also variation between the two systems in the influence of other regional factors on spatial heterogeneity in microbial enzyme activity, including elevation, drainage area, and TWI, as well as factors specific to each habitat, such as organic matter, moisture, and nutrient content. This supports the idea that local habitat heterogeneity primarily governs within-stream variation in microbial activity, while catchment-scale factors exert more control between streams, resulting in a hierarchical and coordinated control of microbial processes in streams [19,65,72].

Ratios of the activity of enzymes involved in carbon, nitrogen, and phosphorus acquisition have been widely used to infer nutrient limitation in stream microbial communities [26,47,73,74,75]. This enzyme stoichiometry approach suggested widespread C and P co-limitation of microbial communities in the different habitats of our study streams, which is consistent with patterns reported for other microbial decomposer communities in streams [4,32,74]. However, within each stream, habitats differed in the occurrence and extent of N limitation, resulting in pronounced spatial variation both within and between the two streams. N limitation was fairly widespread in microbial communities in the water of Pendergrass Creek, whereas only a few water microbial communities showed patterns in enzyme activity suggesting N limitation in Gibson Jack Creek. The opposite pattern was seen for biofilm communities, with more N-limited biofilm communities in Gibson Jack Creek than in Pendergrass Creek. Leaf litter microbial communities showed different patterns in N limitation in each stream: in Gibson Jack Creek, there was widespread N limitation, with localized clusters of C and N co-limitation at several confluences of tributaries and the main channel. N limitation of microbial communities in sediment tended to occur more in tributaries, with Pendergrass Creek exhibiting more clustering of N-limited areas. These spatial differences likely reflect adaptive shifts in microbial nutrient acquisition strategies [57], and as suggested by Kemajou Tchamba et al. [40], such variation may stem from the heterogeneous nature of stream habitats, which are strongly shaped by physicochemical features and environmental conditions [61,76]. Differences between streams likely reflect regional gradients in substrate type, geology, hydrological regime, and latitude [77], but studies on other streams in each region would be needed to confirm this. It should also be noted that while there was substantial spatial variation in nutrient limitation both within and between streams, these inferences are based on enzyme activity measurements at a single time point in summer 2022 at Pendergrass Creek and summer 2023 at Gibson Jack Creek. Whether such nutrient limitation applies throughout an annual cycle or even from year to year would require longer-term studies.

## 5. Conclusions

This study revealed substantial spatial variability in the enzyme activity of microbial communities in different habitats in two intermittent streams. Microbial activity was generally greater in Gibson Jack Creek than in Pendergrass Creek, and while there was high spatial heterogeneity in the activity associated with biofilm, sediment, and water in both systems, leaf litter enzyme activity only showed this heterogeneity in Gibson Jack Creek. Microbial communities in Gibson Jack Creek showed more correlations in enzyme activity between different habitats, while microbial enzyme activity in Pendergrass Creek had stronger within-habitat correlations. Relationships of microbial enzyme activity to environmental conditions were strongest for biofilm and sediment communities in Gibson Jack Creek, where activity was related to concentrations of ions in water, sediment nutrients, organic matter and moisture content, and watershed characteristics, such as elevation, drainage area, and TWI. Stoichiometric analysis of enzyme activities suggested broad co-limitation of microbial communities in both streams by C and P, with spatial variation driven by differences in the occurrence and extent of N limitation. Overall, these patterns show the high variation in microbial activity in different compartments of intermittent streams, and the importance of small- and watershed-scale environmental variables on this activity.

## Figures and Tables

**Table 1:** Sampling information and effective sample size (*n*) by stream habitat and water availability (wet/dry) status.

Stream	Location	Sampling Period	Habitat	Wet Sites (*n*)	Dry Sites (*n*)	Total (*n*)
Gibson Jack Creek *	Idaho, USA	27–30 June 2023	Water	37	0	37
			Biofilm	37	11	48
			Leaf litter	35	11	47
			Sediment	39	11	50
Pendergrass Creek	Alabama, USA	9–10 June 2022	Water	40	0	40
			Biofilm	38	4	42
			Leaf litter	41	6	47
			Sediment	41	6	47

* Sampling period indicates the timing of sample collection for each stream; Wet sites (*n*) and Dry sites (*n*) refer to the number of sites sampled in each stream for each habitat and are designated based on the presence or absence of surface water at the time of sampling, with Total (*n*) being the overall number of sites sampled per habitat.

**Table 2:** Means (±SD) and ranges (*n*) for water chemistry, habitat, and watershed features of the sites sampled at Gibson Jack Creek and Pendergrass Creek.

Variable, Units	Gibson Jack Creek		Pendergrass Creek	
	Mean (±SD)	Range (*n* *)	Mean (±SD)	Range (*n* *)
Water boron (ppm)	0.007 (0.003)	0.0–0.02 (36)	0.007 (0.001)	0.004–0.008 (31)
Water potassium (ppm)	0.4 (0.2)	0.08–0.7 (36)	0.4 (0.1)	0.3–0.5 (31)
Water calcium (ppm)	14.6 (16.4)	2.9–63.2 (36)	0.9 (0.1)	0.6–1.1 (31)
Water magnesium (ppm)	2.5 (1.9)	0.7–9.6 (36)	0.5 (0.1)	0.2–0.7 (31)
Water sodium (ppm)	3.9 (1.4)	1.9–7.9 (36)	1.03 (0.12)	0.9–1.3 (31)
Water silica (ppm)	5.3 (0.9)	3.6–7.3 (36)	3.3 (0.4)	2.4–4.3 (31)
Water strontium (ppm)	0.04 (0.03)	0.013–0.15 (36)	0.004 (0.004)	0.0–0.01 (31)
Water bromide (mgL^−1^)	0.008 (0.002)	0.005–0.013 (29)	0.009 (0.001)	0.006–0.011 (34)
Water chloride (mgL^−1^)	2.7 (1.0)	1.1–4.5 (30)	1.1 (0.1)	0.8–1.2 (34)
Water fluoride (mgL^−1^)	0.03 (0.007)	0.02–0.05 (30)	0.012 (0.003)	0.004–0.02 (34)
Water sulfate (mgL^−1^)	1.9 (0.6)	0.7–3.3 (30)	1.7 (0.4)	0.7–2.3 (34)
Water ammonium (μgL^−1^)	8.9 (4.0)	3.5–24.4 (39)	12.6 (4.7)	2.9–22.7 (39)
Water Nitrate (μgL^−1^)	0.5 (0.7)	0.02–3.3 (30)	0.04 (0.02)	0.02–0.08 (34)
Water non-purgeable organic carbon, NPOC (mgL^−1^)	1.6 (0.5)	0.96–2.7 (39)	1.3 (0.8)	0.3–4.6 (39)
Water Soluble reactive phosphorus, SRP (μgL^−1^)	18.4 (5.1)	9.5–27.1 (39)	5.6 (2.1)	2.1–11.6 (39)
Water Specific conductivity, SPC (μScm^−1^)	121.3 (111.6)	7.0–435.5 (37)	16.5 (1.6)	11.1–19.8 (36)
Stream water temperature (°C)	9.19 (1.53)	7.60–14.10 (37)	19.23 (0.86)	16.60–21.50 (37)
Leaf litter organic matter (AFDM)	0.9 (0.2)	0.0–1.0 (46)	0.9 (0.2)	0.4–1.0 (47)
Leaf litter moisture content	0.1 (0.1)	0.0–0.5 (46)	0.3 (0.1)	0.02–0.5 (47)
Sediment organic matter (AFDM)	0.1 (0.2)	0.01–1.1 (50)	0.04 (0.04)	0.0–0.2 (47)
Sediment ammonium (μgg^−1^)	0.002 (0.002)	0.001–0.01 (50)	0.002 (0.001)	0.001–0.01 (47)
Sediment nitrate (μgg^−1^)	0.02 (0.004)	0.01–0.03 (50)	0.1 (0.02)	0.05–0.1 (47)
Sediment phosphate (μgg^−1^)	0.001 (0.002)	0.004–0.02 (50)	0.008 (0.001)	0.006–0.01 (47)
Sediment moisture content	0.3 (0.1)	0.06–0.6 (50)	0.3 (0.1)	0.2–0.6 (47)
Sediment pH	7.2 (0.4)	6.5–8.1 (50)	6.7 (0.7)	4.8–7.8 (40)
Pressure (mmHg)	617.1 (6.5)	608.1–636.5 (33)	728.6 (2.2)	723.6–731.5 (37)
Topographic wetness index (TWI)	16.4 (1.6)	13.4–19.2 (50)	14.8 (2.1)	11.2–18.1 (47)
Transect width (m)	1.2 (0.7)	0.3–3.8 (48)	1.1 (0.6)	0.4–2.5 (44)
Canopy cover (%)	72.5 (19.1)	33.3–98.7 (50)	92.8 (10.5)	49.0–100.0 (47)
Distance from outlet (km)	3.3 (1.8)	0.0–6.0 (50)	1.3 (0.7)	0.0–2.3 (47)
Drainage area (km^2^)	3.5 (4.3)	0.2–15.3 (50)	0.2 (0.3)	0.01–0.9 (47)
Elevation (m)	1845 (102)	1641–1989 (50)	373 (21)	346–424 (47)

* *n* represents the number of sites sampled, with a potential maximum *n* = 50 for Gibson Jack Creek and *n* = 47 for Pendergrass Creek. Variation in *n* between variables reflects the absence of surface water at some sites during sampling or missing data.

**Table 3:** Mean (±SD) microbial extracellular enzyme activity across habitats in Gibson Jack Creek and Pendergrass Creek.

Habitat	Enzyme	Gibson Jack	Pendergrass	Units
Water	Β-glucosidase	1.23 ± 0.6	0.12 ± 0.13	μmol h^−1^mL^−1^
	Phosphatase	0.94 ± 0.7	1.35 ± 0.45	μmol h^−1^mL^−1^
	N-acetylglucosaminidase	0.06 ± 0.14	0.79 ± 0.5	μmol h^−1^mL^−1^
Biofilm	Β-glucosidase	0.49 ± 0.69	0.05 ± 0.08	μmol h^−1^cm^−2^
	Phosphatase	0.39 ± 0.5	0.13 ± 0.11	μmol h^−1^cm^−2^
	N-acetylglucosaminidase	0.16 ± 0.29	0.06 ± 0.06	μmol h^−1^cm^−2^
Leaf litter	Β-glucosidase	72.01 ± 53.72	31.18 ± 22.47	μmol h^−1^g^−1^
	Phosphatase	55.99 ± 50.45	37.9 ± 21.84	μmol h^−1^g^−1^
	N-acetylglucosaminidase	7.5 ± 7.3	9.17 ± 6.71	μmol h^−1^g^−1^
	Phenol oxidase	15.25 ± 22.68	7.03 ± 5.05	μmol h^−1^g^−1^
	Peroxidase	20.42 ± 35.27	5.45 ± 8.11	μmol h^−1^g^−1^
Sediment	Β-glucosidase	0.58 ± 0.65	0.14 ± 0.16	μmol h^−1^g^−1^
	Phosphatase	0.78 ± 0.69	0.9 ± 0.96	μmol h^−1^g^−1^
	N-acetylglucosaminidase	0.07 ± 0.07	0.05 ± 0.06	μmol h^−1^g^−1^
	Phenol oxidase	0.74 ± 0.54	1.15 ± 0.41	μmol h^−1^g^−1^
	Peroxidase	0.7 ± 0.94	0.67 ± 0.58	μmol h^−1^g^−1^

## Data Availability

Ash-free dry mass data from southeastern forest (AL, USA) seasonal and synoptic stream sampling (AIMS_SE_approach2_approach3_AFDM) presented in the study are openly available in [CUAHSI HydroShare] at [http://www.hydroshare.org/resource/3f0d2d8793834333a5f5d0b185cee857, https://www.hydroshare.org/resource/81c003a7b8474d63a31641a4f375fd18, https://www.hydroshare.org/resource/fac2ee564cf842b8a3ab0e6b30d0d24d, https://www.hydroshare.org/resource/4eb71c9924f54d3a89578caf485aca13, http://www.hydroshare.org/resource/83c327da85f74e50bdf05591b0e1ef82, https://www.hydroshare.org/resource/043f5af4d1ee4ec3aac772c8ae177910, https://www.hydroshare.org/resource/0decb1efb3a34e88b39b64dbb6369743, https://www.hydroshare.org/resource/e36dc69dca0e4fbc969e7ae6137f3744, https://www.hydroshare.org/resource/dc0434b19c834941aa56449af0f6ce9b, https://www.hydroshare.org/resource/e80e4db42de940aa9fe18667dddebec4, https://www.hydroshare.org/resource/280690c6068c438ea62a46d5b0c68233, https://www.hydroshare.org/resource/eb7624d386584c1fb5468ed376487552, https://www.hydroshare.org/resource/e2cf9b794cfa449b9abca5fe1ea2848d/, https://www.hydroshare.org/resource/a38c039c409b48e7b37c9bfabf5e3481/, https://www.hydroshare.org/resource/4e954f5b05d34ab3a58005b76fd5a49a/].

## Supplementary Materials

The following supporting information can be downloaded at https://www.mdpi.com/article/10.3390/microorganisms14010071/s1: Figure S1: Kendal’s tau correlation between microbial enzyme activity in Gibson Jack; Figure S2: Forest plot showing the effect of water chemistry on the spatial variation in enzyme activity across habitats—sediment (diamonds), leaf litter (triangles), biofilm (squares), and water (circles)—in Gibson Jack (red) and Pendergrass (blue); Figure S3: Forest plot showing the effect of watershed and habitat characteristics on the spatial variation in enzyme activity in biofilm (squares) and sediment (diamonds) at Gibson Jack (red) and Pendergrass (blue); Figure S4: Spatial distribution of microbial nutrient limitation based on combined hydrolase–oxidase stoichiometry in leaf litter and sediment across 50 sites in Gibson Jack Creek (**a**,**b**) and 47 sites in Pendergrass Creek (**c**,**d**); Table S1: Summary of generalized additive models (GAMs) examining spatial differences in enzyme activity in water and epilithic biofilm, leaf litter, and sediment using geographical coordinates (latitude, longitude) and in-stream distances as smooth terms.

## Abbreviations

The following abbreviations are used in this manuscript:

BG	Β-glucosidase
P	Phosphatase
NAG	N-acetylglucosaminidase
POX	Phenol oxidase
PER	Peroxidase
pNP	4-nitrophenyl
DOPA	L-3,4-dihydroxyphenylalanine
MUB	4-methylumbelliferone
pRDA	Partial redundancy analysis
IQR	Interquartile range
FDR	False discovery rate
SD	Standard deviation
GWR	Geographically weighted regression
rpm	Rotation per minute
DOC	Dissolved organic carbon
DEM	Digital elevation model
TWI	Topographic wetness index
STIC	Stream Temperature Intermittency Conductivity
TOC	Total organic carbon
TDN	Total dissolved nitrogen
CH_4_	Methane
CO_2_	Carbon dioxide
N	Nitrogen
P	Phosphorus
C	Carbon
POC	Particulate organic carbon
AFDM	Ash-free dry mass

## References

[1] Swan C., Boyero L., Canhoto C. (2021). The Ecology of Plant Litter Decomposition in Stream Ecosystems.

[2] Arnosti C., Bell C., Moorhead D.L., Sinsabaugh R.L., Steen A.D., Stromberger M., Wallenstein M., Weintraub M.N. (2014). Extracellular Enzymes in Terrestrial, Freshwater, and Marine Environments: Perspectives on System Variability and Common Research Needs. Biogeochemistry.

[3] Romaní A.M., Chauvet E., Febria C., Mora-Gómez J., Risse-Buhl U., Timoner X., Weitere M., Zeglin L., Datry T., Bonada N., Boulton A. (2017). Chapter 4.1—The Biota of Intermittent Rivers and Ephemeral Streams: Prokaryotes, Fungi, and Protozoans. Intermittent Rivers and Ephemeral Streams.

[4] Johnson L.T., Tank J.L., Dodds W.K. (2009). The Influence of Land Use on Stream Biofilm Nutrient Limitation across Eight North American Ecoregions. Can. J. Fish. Aquat. Sci..

[5] Bernot M.J., Sobota D.J., Hall R.O., Mulholland P.J., Dodds W.K., Webster J.R., Tank J.L., Ashkenas L.R., Cooper L.W., Dahm C.N. (2010). Inter-Regional Comparison of Land-Use Effects on Stream Metabolism. Freshw. Biol..

[6] Shumilova O., Zak D., Datry T., von Schiller D., Corti R., Foulquier A., Obrador B., Tockner K., Allan D.C., Altermatt F. (2019). Simulating Rewetting Events in Intermittent Rivers and Ephemeral Streams: A Global Analysis of Leached Nutrients and Organic Matter. Glob. Change Biol..

[7] Schilling O.S., Cook P.G., Grierson P.F., Dogramaci S., Simmons C.T. (2021). Controls on Interactions Between Surface Water, Groundwater, and Riverine Vegetation Along Intermittent Rivers and Ephemeral Streams in Arid Regions. Water Resour. Res..

[8] Costigan K.H., Jaeger K.L., Goss C.W., Fritz K.M., Goebel P.C. (2016). Understanding Controls on Flow Permanence in Intermittent Rivers to Aid Ecological Research: Integrating Meteorology, Geology and Land Cover. Ecohydrology.

[9] Datry T., Corti R., Heino J., Hugueny B., Rolls R.J., Ruhí A., Datry T., Bonada N., Boulton A. (2017). Chapter 4.9—Habitat Fragmentation and Metapopulation, Metacommunity, and Metaecosystem Dynamics in Intermittent Rivers and Ephemeral Streams. Intermittent Rivers and Ephemeral Streams.

[10] Raymond P.A., Saiers J.E., Sobczak W.V. (2016). Hydrological and Biogeochemical Controls on Watershed Dissolved Organic Matter Transport: Pulse-Shunt Concept. Ecology.

[11] Datry T., Larned S.T., Tockner K. (2014). Intermittent Rivers: A Challenge for Freshwater Ecology. BioScience.

[12] Acuña V., Datry T., Marshall J., Barceló D., Dahm C.N., Ginebreda A., McGregor G., Sabater S., Tockner K., Palmer M.A. (2014). Why Should We Care About Temporary Waterways?. Science.

[13] Tooth S., Nanson G.C. (2011). Distinctiveness and Diversity of Arid Zone River Systems. Arid Zone Geomorphology.

[14] Buttle J.M., Boon S., Peters D.L., Spence C., van Meerveld H.J., Whitfield P.H. (2012). An Overview of Temporary Stream Hydrology in Canada. Can. Water Resour. J. Rev. Can. Ressour. Hydr..

[15] von Schiller D., Bernal S., Dahm C.N., Martí E., Datry T., Bonada N., Boulton A. (2017). Chapter 3.2—Nutrient and Organic Matter Dynamics in Intermittent Rivers and Ephemeral Streams. Intermittent Rivers and Ephemeral Streams.

[16] Pisani O., Dodds W.K., Jaffé R. (2016). Characterizing Organic Matter Inputs to Sediments of Small, Intermittent, Prairie Streams: A Molecular Marker and Stable Isotope Approach. Aquat. Sci..

[17] Bruder A., Chauvet E., Gessner M.O. (2011). Litter Diversity, Fungal Decomposers and Litter Decomposition under Simulated Stream Intermittency. Funct. Ecol..

[18] Ademollo N., Capri S., Patrolecco L., Puddu A., Polesello S., Rusconi M., Valsecchi S., Froebrich J. (2011). Fate and Monitoring of Hazardous Substances in Temporary Rivers. TrAC Trends Anal. Chem..

[19] Zeglin L.H. (2015). Stream Microbial Diversity in Response to Environmental Changes: Review and Synthesis of Existing Research. Front. Microbiol..

[20] Dodds W.K., Gido K., Whiles M.R., Daniels M.D., Grudzinski B.P. (2015). The Stream Biome Gradient Concept: Factors Controlling Lotic Systems across Broad Biogeographic Scales. Freshw. Sci..

[21] Dodds W.K., Higgs S.A., Spangler M.J., Guinnip J., Scott J.D., Hedden S.C., Frenette B.D., Taylor R., Schechner A.E., Hoeinghaus D.J. (2018). Spatial Heterogeneity and Controls of Ecosystem Metabolism in a Great Plains River Network. Hydrobiologia.

[22] Price A.N., Jones C.N., Hammond J.C., Zimmer M.A., Zipper S.C. (2021). The Drying Regimes of Non-Perennial Rivers and Streams. Geophys. Res. Lett..

[23] Mosher J.J., Kaplan L.A., Podgorski D.C., McKenna A.M., Marshall A.G. (2015). Longitudinal Shifts in Dissolved Organic Matter Chemogeography and Chemodiversity within Headwater Streams: A River Continuum Reprise. Biogeochemistry.

[24] Findlay S., Jones J.B., Stanley E.H. (2016). Chapter 3—Stream Microbial Ecology in a Changing Environment. Stream Ecosystems in a Changing Environment.

[25] Sinsabaugh R.L., Lauber C.L., Weintraub M.N., Ahmed B., Allison S.D., Crenshaw C., Contosta A.R., Cusack D., Frey S., Gallo M.E. (2008). Stoichiometry of Soil Enzyme Activity at Global Scale. Ecol. Lett..

[26] Hill B.H., Elonen C.M., Seifert L.R., May A.A., Tarquinio E. (2012). Microbial Enzyme Stoichiometry and Nutrient Limitation in US Streams and Rivers. Ecol. Indic..

[27] Findlay S., Strayer D., Goumbala C., Gould K. (1993). Metabolism of Streamwater Dissolved Organic Carbon in the Shallow Hyporheic Zone. Limnol. Oceanogr..

[28] Battin T.J., Kaplan L.A., Denis Newbold J., Hansen C.M.E. (2003). Contributions of Microbial Biofilms to Ecosystem Processes in Stream Mesocosms. Nature.

[29] Romaní A.M., Sabater S. (2001). Structure and Activity of Rock and Sand Biofilms in a Mediterranean Stream. Ecology.

[30] Hill B.H., McCormick F.H., Harvey B.C., Johnson S.L., Warren M.L., Elonen C.M. (2010). Microbial Enzyme Activity, Nutrient Uptake and Nutrient Limitation in Forested Streams. Freshw. Biol..

[31] Frossard A., Gerull L., Mutz M., Gessner M.O. (2012). Disconnect of Microbial Structure and Function: Enzyme Activities and Bacterial Communities in Nascent Stream Corridors. ISME J..

[32] Sinsabaugh R.L., Follstad Shah J.J., Hill B.H., Elonen C.M. (2012). Ecoenzymatic Stoichiometry of Stream Sediments with Comparison to Terrestrial Soils. Biogeochemistry.

[33] Bullock A., Ziervogel K., Ghobrial S., Smith S., McKee B., Arnosti C. (2017). A Multi-Season Investigation of Microbial Extracellular Enzyme Activities in Two Temperate Coastal North Carolina Rivers: Evidence of Spatial but Not Seasonal Patterns. Front. Microbiol..

[34] Romaní A.M., Giorgi A., Acuña V., Sabater S. (2004). The Influence of Substratum Type and Nutrient Supply on Biofilm Organic Matter Utilization in Streams. Limnol. Oceanogr..

[35] Dohman J.M., Godsey S.E., Hale R.L. (2021). Three-Dimensional Subsurface Flow Path Controls on Flow Permanence. Water Resour. Res..

[36] Rodgers D., Long S., McQuarrie N., Burgel W.D., Hersley C.F. (2006). Geologic Map of the Inkom Quadrangle, Bannock County, Idaho.

[37] Williams J., Bogan A., Garner J. (2008). Freshwater Mussels of Alabama and the Mobile Basin in Georgia, Mississippi and Tennessee.

[38] Plont S., Peterson D.M., Smith C.R., Bond C.T., Tchamba A.L.K., Wolford M.A., Zarek K., Speir S.L., Jones C.N., Benstead J.P. (2025). Hydrologic, Biogeochemical, Microbial, and Macroinvertebrate Responses to Network Expansion, Contraction, and Disconnection across Headwater Stream Networks with Distinct Physiography in Alabama, USA. Earth Syst. Sci. Data Discuss..

[39] Griffith G.A., Omernik J., Comstock J.A., Lawrence S., Martin G., Goddard A., Hulcher V.J., Foster T. Ecoregions of Alabama and Georgia, (Color Poster with Map, Descriptive Text, Summary Tables, and Photographs). https://www.epa.gov/eco-research/ecoregion-download-files-state-region-4.

[40] Kemajou Tchamba A.L., Bond C.T., Nave B.A., Ramos R., Seybold E.C., Burgin A.J., Aho K., You Y., Zeglin L.H., Kuehn K.A. (2025). Spatial Variation in Microbial Enzyme Activity in an Intermittent Stream Network. Discov. Water.

[41] Bond C.T., Nave B.A., Kemajou Tchamba A.L., Stanley E., Zeglin L.H., Jackson C.R., Zipper S., Aho K., Burgin A.J., You Y. (2025). Fungal Communities across a Surface Water Permanence Gradient in a Non-Perennial Prairie Stream Network. ISME Commun..

[42] Wu Q., Brown A. (2024). Whitebox: “WhiteboxTools” R Frontend 2024.

[43] Chapin T.P., Todd A.S., Zeigler M.P. (2014). Robust, Low-Cost Data Loggers for Stream Temperature, Flow Intermittency, and Relative Conductivity Monitoring. Water Resour. Res..

[44] Hood-Nowotny R., Umana N.H.-N., Inselbacher E., Oswald- Lachouani P., Wanek W. (2010). Alternative Methods for Measuring Inorganic, Organic, and Total Dissolved Nitrogen in Soil. Soil Sci. Soc. Am. J..

[45] Ringuet S., Sassano L., Johnson Z.I. (2011). A Suite of Microplate Reader-Based Colorimetric Methods to Quantify Ammonium, Nitrate, Orthophosphate and Silicate Concentrations for Aquatic Nutrient Monitoring. J. Environ. Monit..

[46] Jackson C.R., Tyler H.L., Millar J.J. (2013). Determination of Microbial Extracellular Enzyme Activity in Waters, Soils, and Sediments Using High Throughput Microplate Assays. J. Vis. Exp. JoVE.

[47] Moorhead D.L., Sinsabaugh R.L., Hill B.H., Weintraub M.N. (2016). Vector Analysis of Ecoenzyme Activities Reveal Constraints on Coupled C, N and P Dynamics. Soil Biol. Biochem..

[48] R Core Team (2024). R: A Language and Environment for Statistical Computing.

[49] Wei T., Simko V., Levy M., Xie Y., Jin Y., Zemla J., Freidank M., Cai J., Protivinsky T. (2024). Corrplot: Visualization of a Correlation Matrix.

[50] Wood S.N., Pya N., Säfken B. (2016). Smoothing Parameter and Model Selection for General Smooth Models. J. Am. Stat. Assoc..

[51] Oksanen J., Simpson G.L., Blanchet F.G., Kindt R., Legendre P., Minchin P.R., O’Hara R.B., Solymos P., Stevens M.H.H., Szoecs E. (2025). Vegan: Community Ecology Package.

[52] Lu B., Harris P., Charlton M., Brunsdon C. (2014). The GWmodel R Package: Further Topics for Exploring Spatial Heterogeneity Using Geographically Weighted Models. Geo-Spat. Inf. Sci..

[53] Gollini I., Lu B., Charlton M., Brunsdon C., Harris P. (2015). GWmodel: An R Package for Exploring Spatial Heterogeneity Using Geographically Weighted Models. J. Stat. Softw..

[54] Romaní A.M., Amalfitano S., Artigas J., Fazi S., Sabater S., Timoner X., Ylla I., Zoppini A. (2013). Microbial Biofilm Structure and Organic Matter Use in Mediterranean Streams. Hydrobiologia.

[55] Pohlon E., Fandino A.O., Marxsen J. (2013). Bacterial Community Composition and Extracellular Enzyme Activity in Temperate Streambed Sediment during Drying and Rewetting. PLoS ONE.

[56] Williamson T.J., Vanni M.J., Renwick W.H. (2021). Spatial and Temporal Variability of Nutrient Dynamics and Ecosystem Metabolism in a Hyper-Eutrophic Reservoir Differ Between a Wet and Dry Year. Ecosystems.

[57] Hill B.H., Elonen C.M., Jicha T.M., Bolgrien D.W., Moffett M.F. (2010). Sediment Microbial Enzyme Activity as an Indicator of Nutrient Limitation in the Great Rivers of the Upper Mississippi River Basin. Biogeochemistry.

[58] Chen X., Xu G., Fang K., Wan S., Gu F., Wang B., Li J., Cheng Y. (2025). Microbial Diversity Patterns and Extracellular Enzyme Activities of River Sediment in Different Geomorphic Regions of the Loess Plateau. Environ. Earth Sci..

[59] Zhang M., Cheng X., Geng Q., Shi Z., Luo Y., Xu X. (2019). Leaf Litter Traits Predominantly Control Litter Decomposition in Streams Worldwide. Glob. Ecol. Biogeogr..

[60] Omoniyi G.E., Bergerot B., Pellan L., Delmotte M., Crave A., Heyman J., Piscart C. (2021). In-Stream Variability of Litter Breakdown and Consequences on Environmental Monitoring. Water.

[61] Bastias E., Sponseller R.A., Bundschuh M., Jonsson M. (2022). Seasonal Variation in the Coupling of Microbial Activity and Leaf Litter Decomposition in a Boreal Stream Network. Freshw. Biol..

[62] del Campo R., Blackman R.C., Martini J., Fuß T., Thuile Bistarelli L., Gessner M.O., Altermatt F., Singer G. (2025). Functional Macroinvertebrate Diversity Stabilizes Decomposition among Leaf Litter Resources across a River Network. Ecol. Monogr..

[63] Rier S.T., Nawrocki K.S., Whitley J.C. (2011). Response of Biofilm Extracellular Enzymes along a Stream Nutrient Enrichment Gradient in an Agricultural Region of North Central Pennsylvania, USA. Hydrobiologia.

[64] Martyniuk N., Souza M.S., Bastidas Navarro M., Balseiro E., Modenutti B. (2022). Nutrient Limitation Affects Biofilm Enzymatic Activities in a Glacier-Fed River. Hydrobiologia.

[65] Bastias E., Bolivar M., Ribot M., Peipoch M., Thomas S.A., Sabater F., Martí E. (2020). Spatial Heterogeneity in Water Velocity Drives Leaf Litter Dynamics in Streams. Freshw. Biol..

[66] Bier R.L., Mosher J.J., Kaplan L.A., Kan J. (2023). Spatial Scale Impacts Microbial Community Composition and Distribution within and across Stream Ecosystems in North and Central America. Environ. Microbiol..

[67] Matulich K.L., Weihe C., Allison S.D., Amend A.S., Berlemont R., Goulden M.L., Kimball S., Martiny A.C., Martiny J.B. (2015). Temporal Variation Overshadows the Response of Leaf Litter Microbial Communities to Simulated Global Change. ISME J..

[68] Hale R.L., Godsey S.E. (2019). Dynamic Stream Network Intermittence Explains Emergent Dissolved Organic Carbon Chemostasis in Headwaters. Hydrol. Process..

[69] Peterson D.M., Jones C.N., Zarek K., Wolford M., Smith C., Bond C.T., Plont S., Kraft M., Speir S., Zipper S. (2025). Using Physiography as a Lens to Understand Stream Network Expansion and Contraction Across Spatiotemporal Scales.

[70] Mosher J.J., Findlay R.H. (2011). Direct and Indirect Influence of Parental Bedrock on Streambed Microbial Community Structure in Forested Streams. Appl. Environ. Microbiol..

[71] Nelson M.L., Rhoades C.C., Dwire K.A. (2011). Influence of Bedrock Geology on Water Chemistry of Slope Wetlands and Headwater Streams in the Southern Rocky Mountains. Wetlands.

[72] Mosher J.J., Klein G.C., Marshall A.G., Findlay R.H. (2010). Influence of Bedrock Geology on Dissolved Organic Matter Quality in Stream Water. Org. Geochem..

[73] Wisnoski N.I., Lennon J.T. (2021). Microbial Community Assembly in a Multi-Layer Dendritic Metacommunity. Oecologia.

[74] Hill B.H., Elonen C.M., Herlihy A.T., Jicha T.M., Serenbetz G. (2018). Microbial Ecoenzyme Stoichiometry, Nutrient Limitation, and Organic Matter Decomposition in Wetlands of the Conterminous United States. Wetl. Ecol. Manag..

[75] Cui Y., Bing H., Fang L., Jiang M., Shen G., Yu J., Wang X., Zhu H., Wu Y., Zhang X. (2021). Extracellular Enzyme Stoichiometry Reveals the Carbon and Phosphorus Limitations of Microbial Metabolisms in the Rhizosphere and Bulk Soils in Alpine Ecosystems. Plant Soil.

[76] Gao G., Li G., Liu M., Li P., Liu J., Ma S., Li D., Petropoulos E., Wu M., Li Z. (2023). Changes in Soil Stoichiometry, Soil Organic Carbon Mineralization and Bacterial Community Assembly Processes across Soil Profiles. Sci. Total Environ..

[77] Graça M.A.S., Poquet J.M. (2014). Do Climate and Soil Influence Phenotypic Variability in Leaf Litter, Microbial Decomposition and Shredder Consumption?. Oecologia.

