# Supplementary material for: Streambed Microbial Activity and Its Spatial Distribution in Two Intermittent Stream Networks
Source: Microorganisms. 2025 Dec 29;14(1):71. doi: 10.3390/microorganisms14010071 (PMC12844215; doi:10.3390/microorganisms14010071)
Supplement: Supplementary file 1 [file microorganisms-14-00071-s001.zip › Supplementary Information_S2.pdf]

Supplementary Information

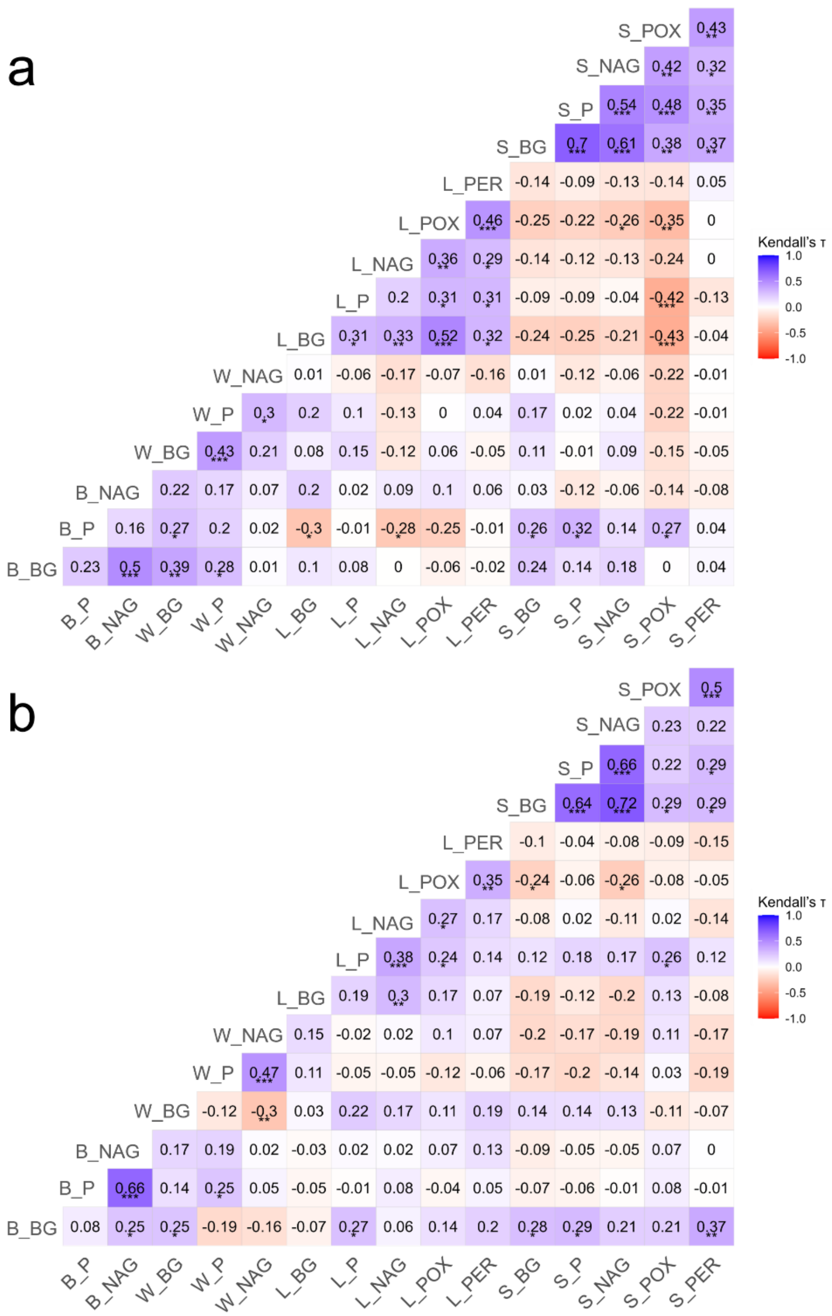

Figure S1. Kendal's tau correlation between microbial enzyme activity in Gibson Jack (a) and Pendergrass (b) streambed habitats: water (W), biofilm (B), leaf litter (L) and sediment (S). Then enzymes are β-glucosidase (BG), phosphatase (P), N-acetylglucosaminidase (NAG), phenol oxidase (POX) and peroxidase (PER). Enzyme activity was denoted by habitat followed by the enzyme abbreviation; for example B_BG

represents β-glucosidase activity in biofilm. Positive (blue) and negative (red) correlations shown, where asterisks indicate significance (*p<0.05, **p<0.01, ***p<0.001). Effective sample sizes (n) represent complete observations used in the analysis and varied by habitat and stream: water – Gibson Jack Creek n = 35, Pendergrass Creek n = 40; biofilm – Gibson Jack Creek n = 48, Pendergrass Creek n = 42, leaf litter – Gibson Jack Creek n = 45, Pendergrass Creek n = 47; sediment – Gibson Jack Creek n = 50, Pendergrass Creek n = 47

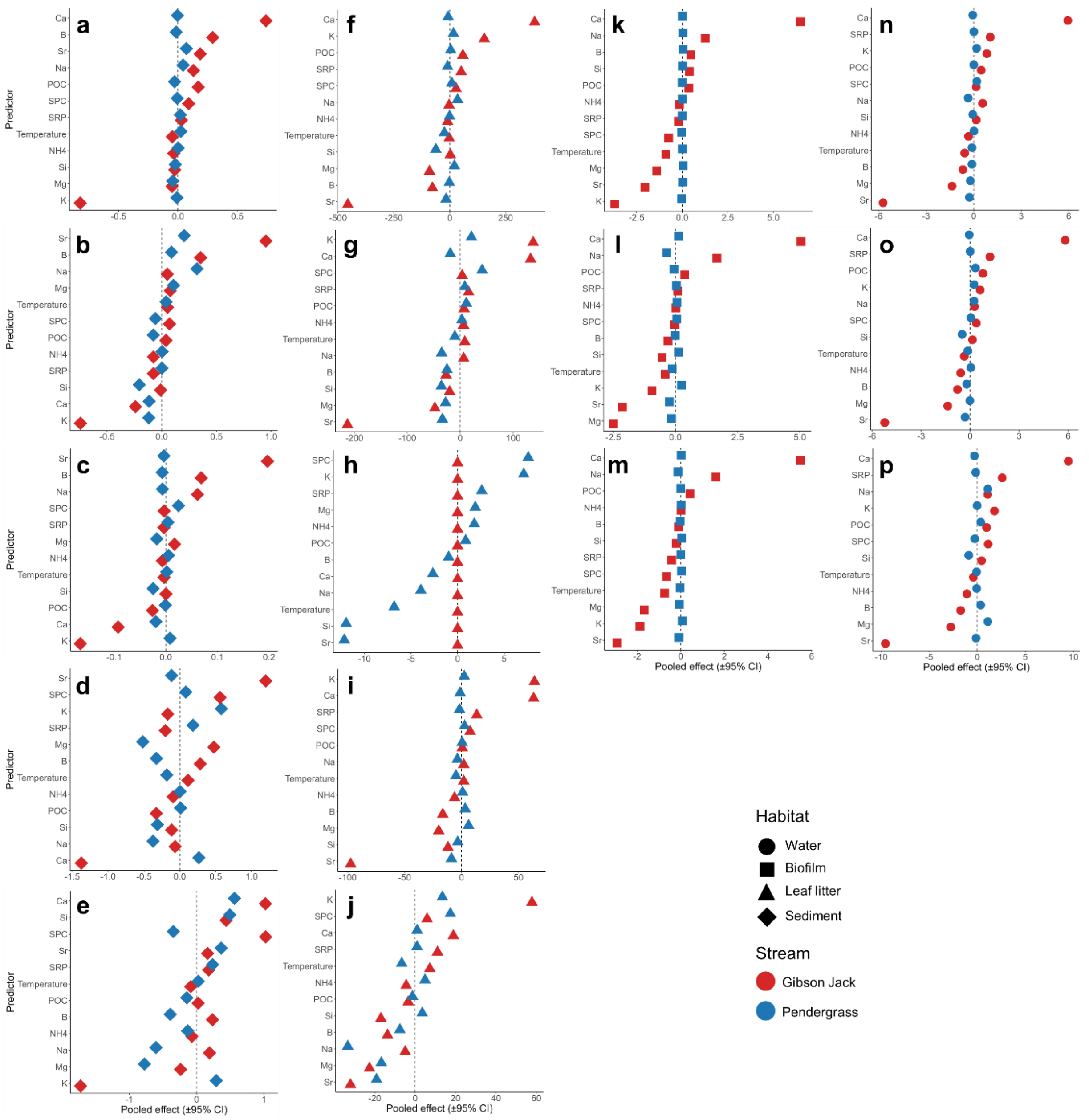

Figure S2. Forest plot showing the effect of water chemistry on the spatial variation of enzyme activity across habitats - sediment (diamonds), leaf litter (triangles), biofilm (squares) and water (circles) - in Gibson Jack (red; n =22) and Pendergrass (blue; n = 27). Panels represent β-glucosidase (a, f, k, n), phosphatase (b, g, l, o), N-acetylglucosaminidase (c, h, m, p), phenol oxidase (d, i) and peroxidase (e, j). Points denote standardized effect sizes (pooled coefficients) with 95 % confidence interval; values right of the vertical line (0) indicate positive effects on enzyme activity, and those left indicate negative effects

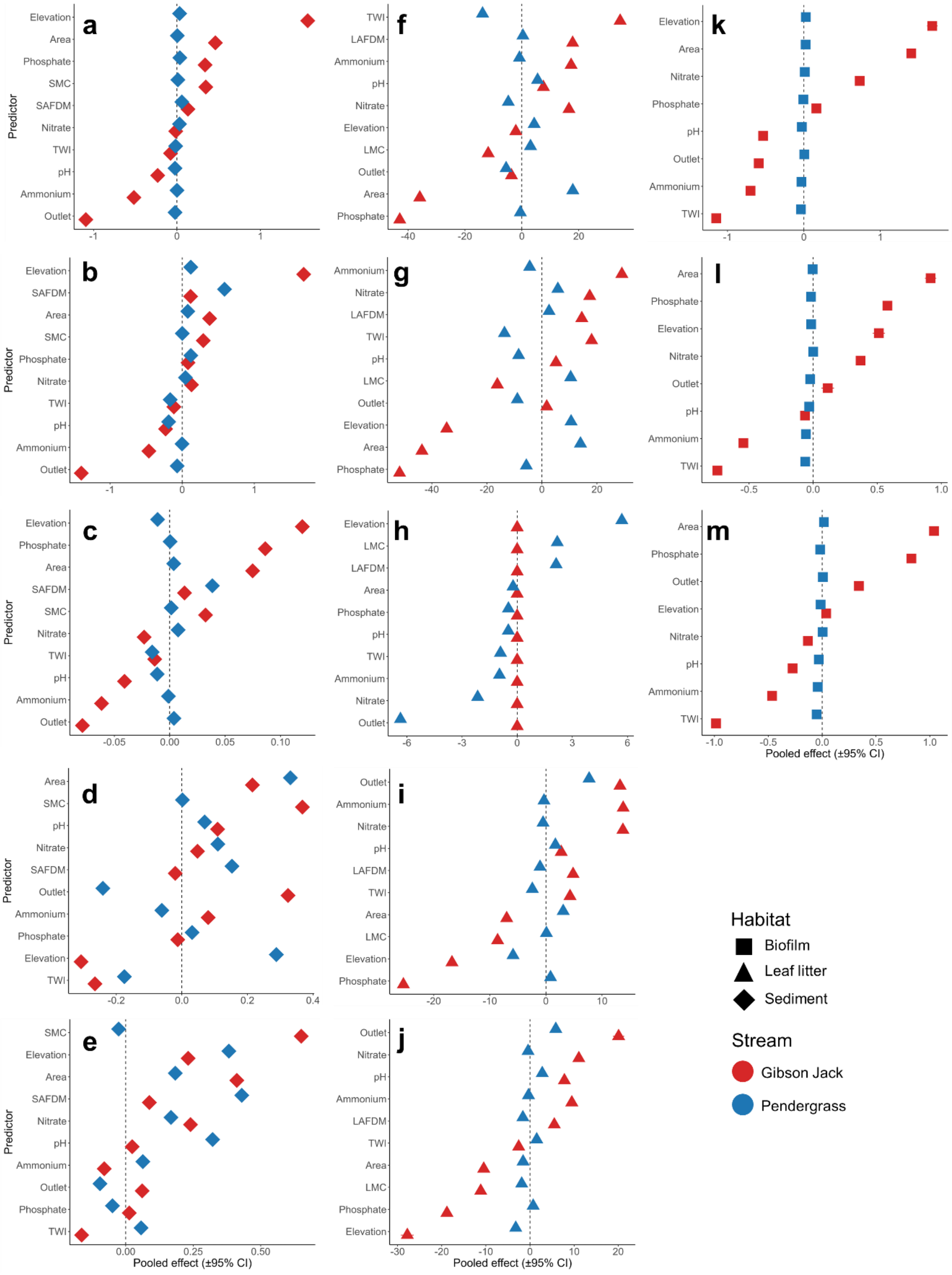

Figure S3. Forest plot showing the effect of watershed and habitat characteristics on the spatial variation of enzyme activity in biofilm (squares) and sediment (diamonds) at

Gibson Jack (red; n =41) and Pendergrass (blue; n = 35). Activities of β-glucosidase (a, d), phosphatase (b, e), N-acetylglucosaminidase (c, f), phenol oxidase (g) and peroxidase (h). Points represent standardized effect sizes (pooled coefficients) with 95 % confidence interval. Values to the right of the vertical dotted line (0) indicate positive effects on enzyme activity, while values to the left indicate negative effects.

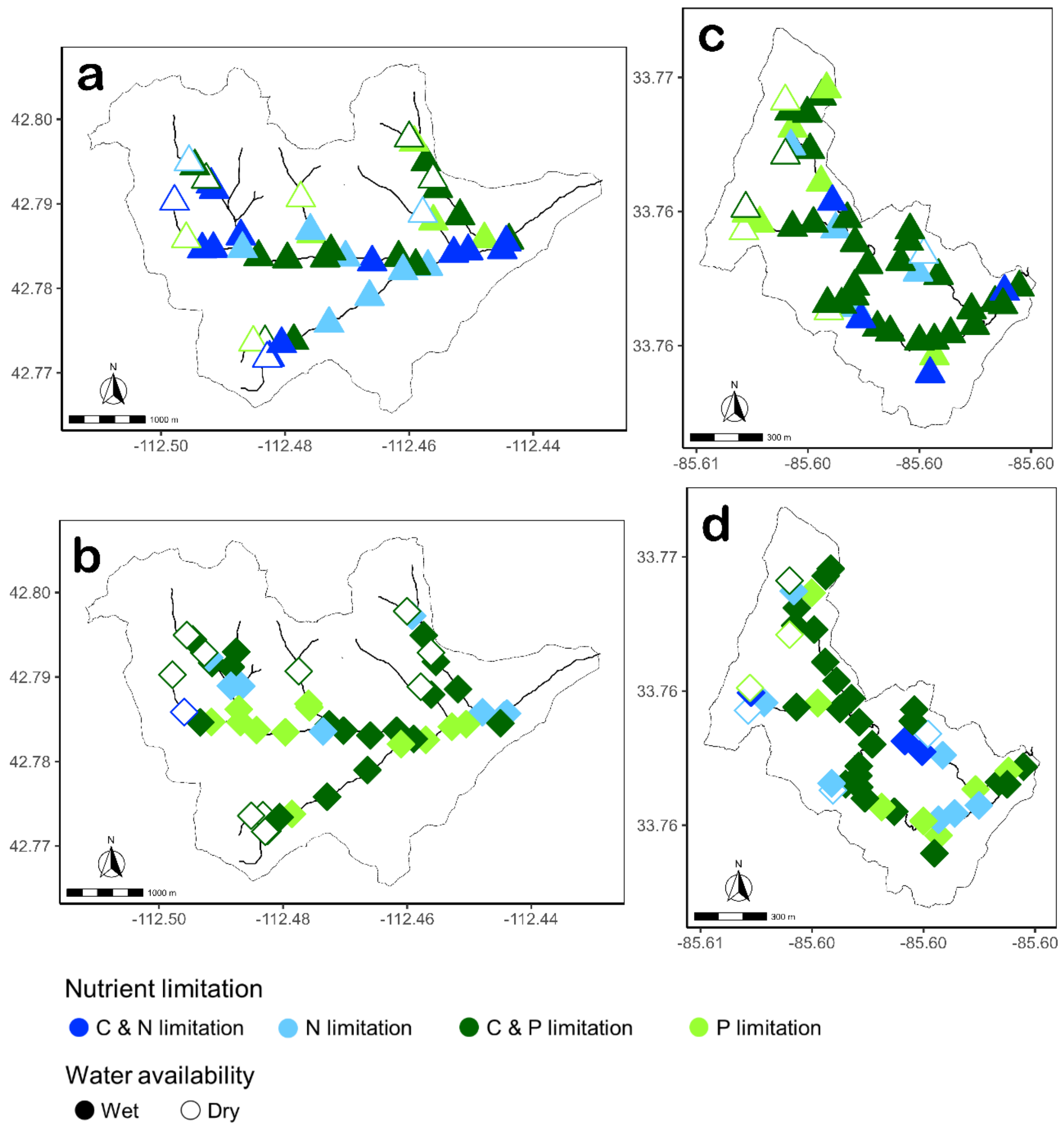

Figure S4. Spatial distribution of microbial nutrient limitation based on combined hydrolase-oxidase stoichiometry in leaf litter and sediment across 50 sites in Gibson Jack Creek (a-b) and 47sites at Pendergrass Creek (c-d). Nutrient limitation was categorized into four groups: N limited (light blue), P limited (light green), C and N co-limited (dark blue) and C and P co-limited (dark green). Filled symbols represent wet sites at Gibson Jack (n=41) and Pendergrass (n = 39); open symbols represent dry sites (n= 11 and 6 respectively) at the time of sampling
